# A Prescribed Digital Health App and Number of Migraine Days: A Randomized Clinical Trial

**DOI:** 10.1001/jamanetworkopen.2025.17708

**Published:** 2025-07-01

**Authors:** Daniel Pach, Stefanie Lysk, Priska Heinz, Ulrike Held, Elif Huber, Simon Scholler, Markus A. Dahlem, Martin Lysk, Jürgen Barth, Katja Icke, Claudia M. Witt

**Affiliations:** 1 Charité*–*Universitätsmedizin Berlin, corporate member of Freie Universität Berlin and Humboldt-Universität zu Berlin, Institute of Social Medicine, Epidemiology and Health Economics, Berlin, Germany; 2 Newsenselab GmbH, Berlin, Germany; 3 Department of Biostatistics at Epidemiology, Biostatistics and Prevention Institute, University of Zurich, Zurich, Switzerland; 4 Robert Koch Institute, Berlin, Germany; 5 Migraine Aura Foundation, Berlin, Germany; 6 Institute for Complementary and Integrative Medicine, University Hospital Zurich and University Zurich, Zurich, Switzerland

## Abstract

**Importance:**

The digital therapeutics market is expanding rapidly, with growing patient adoption. In some countries, digital health applications are already prescribable, reshaping clinical practice.

**Objective:**

To compare the effectiveness of the prescribable digital health app used in this study with a control app in reducing the number of migraine days among adults diagnosed with migraine.

**Design, Setting, and Participants:**

This 2-arm, parallel-group randomized clinical trial was conducted remotely across Germany from March to December 2021. Adults (≥18 years of age) with episodic or chronic migraine were randomly assigned in a 1:1 ratio to the intervention (n = 238) using a prescribable digital health app, called M-sense Migräne, or to the control (n = 239) group. Data were analyzed from February to March 2022.

**Intervention:**

The intervention group used the full version of the prescribable digital health app, while the control group used an app version limited to headache diary features.

**Main Outcomes and Measures:**

The primary outcome was the number of migraine days per a 28-day month after 12 weeks of using the app (assessed weeks 9-12), analyzed using analysis of covariance adjusted for baseline migraine days and migraine type. Secondary outcomes included the number of headache days per month, quality of life (assessed with the 6-item Headache Impact Test), self-efficacy (assessed with the Headache Management Self-Efficacy Scale German Short Form), Headache-Attributed Lost Time in the previous 30 days (HALT-30), migraine-specific health literacy, and days with acute headache medication use.

**Results:**

Of 477 randomized participants, 476 were included in the intention-to-treat population (intervention, n = 238; control, n = 238). The participants’ overall mean (SD) age was 35.2 (10.4) years, 429 were female (90.1%); and the mean (SD) migraine history was 17.42 (10.49) years. After 12 weeks, there was no significant difference in the number of migraine days per month between groups. Migraine days per month decreased from a mean (SD) of 7.74 (3.70) to an adjusted mean of 5.79 (95% CI, 5.02-6.57) in the intervention group and from a mean (SD) of 7.76 (3.69) to an adjusted mean of 5.41 (95% CI, 4.63-6.19) in the control group, with an estimated between-group difference of 0.38 days (95% CI, −0.32 to 1.08 days; *P* = .29). There were no group differences for most secondary outcomes. No serious adverse reactions were reported.

**Conclusions and Relevance:**

In this randomized clinical trial, the prescribed digital migraine health app did not show superiority in reducing migraine days compared with a control app having only basic headache documentation features. This finding underscores the need for randomized clinical trials as a cornerstone of a robust evaluation framework, ensuring both treatment efficacy and patient safety in an expanding digital therapeutics market.

**Trial Registration:**

German Clinical Trials Register (DRKS) identifier: DRKS00024174

## Key Points

**Question:**

Is a prescribed mobile app more effective than a control app in reducing the number of migraine days among patients with migraine headaches in Germany?

**Findings:**

In this randomized clinical trial of 477 patients with migraine, the prescribable digital health app did not demonstrate a superior reduction in the number of migraine days compared with a control app that provided only basic documentation features during a 12-week period.

**Meaning:**

The findings suggest that even if promising observational data exist for digital therapeutics, robust randomized clinical trials are needed to evaluate the effectiveness.

## Teaser

This randomized clinical trial compares the effectiveness of a prescribable digital health app with a control app to reduce the number of migraine days among adults diagnosed with migraine.

## Introduction

The digital therapeutics market is rapidly expanding, with a substantial increase in patient uptake expected in the coming years. In some countries, digital health applications are already prescribable, transforming clinical practices and patient care pathways. Since 2020, German physicians and psychotherapists have been able to prescribe digital health applications (DiGA) that were approved by the German Federal Institute for Drugs and Medical Devices.^1^ Applications (apps) must meet the DiGA-requirements^1^ concerning safety, data protection, quality, patient-centered positive health care effects, and interoperability and be classified and approved as a low-risk medical device. Although many apps lack comprehensive effectiveness evaluations, they may be provisionally listed and reimbursed if they provide systematic data that indicate positive health care benefits, with all DiGA standards^1,2^ met. During a 12-month fast-track period, apps are reimbursed but must undergo evaluation against a comparator group that is supervised and approved by the German Federal Institute for Drugs and Medical Devices.

Migraine affects 12% of the general population, predominantly women.^3^ Management includes both pharmacological and nonpharmacological approaches, with clinical guidelines^4,5,6^ recommending preventive treatments and the use of headache diaries for patients experiencing a minimum of 2 or 3 migraine attacks per month. Among nonpharmacological therapies, relaxation techniques, endurance sports, behavioral therapy on stress reduction, and trigger management are recommended.

Advancements in digital technology have introduced apps to aid migraine management, ranging from electronic diaries to more comprehensive apps integrating behavioral advice.^7^ These apps are increasingly popular for their tracking capabilities and personalized features, although randomized clinical trials (RCTs) to confirm their effectiveness are limited.^8,9,10,11^ This study investigated the one of the most frequently downloaded commercially available headache apps in Germany,^12^ during its 12-month DiGA evaluation phase. Previous observational data assessing this study’s prescribable digital health app suggested promising results.^13^ The present study evaluated the effectiveness of the prescribable digital health app compared with an app version limited to headache diary features in reducing migraine days among patients with migraine.

## Methods

### Study Design

**Figure 1.**
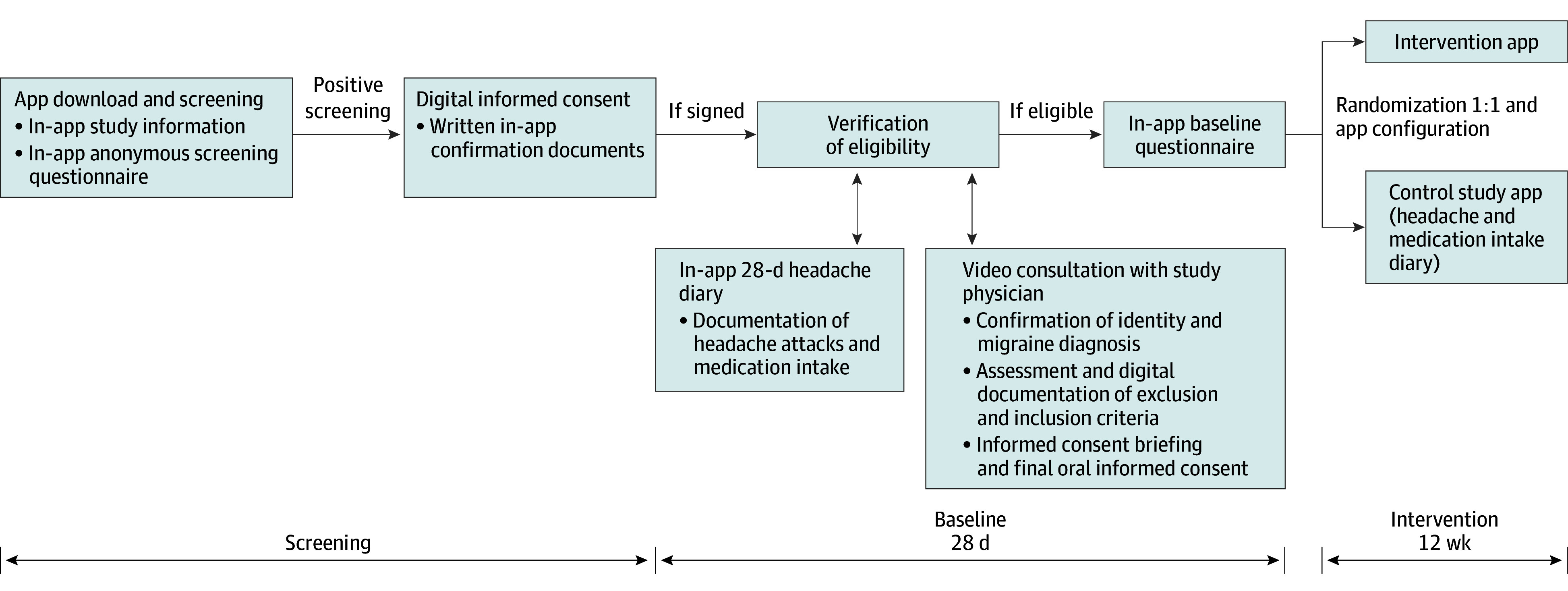
Study Design for the Remote Trial

We report herein the results of a fully remote, app-based, 2-arm, open-label, parallel-group RCT (trial protocol in Supplement 1). This study followed the recommendations of the International Headache Society for controlled clinical trials in episodic^14^ and chronic^15^ migraine and was approved by the Charité–Universitätsmedizin Berlin ethics committee. Findings were reported using the Consolidated Standards of Reporting Trials (CONSORT)^16^ reporting guideline. Potential study participants could download the app, in which they received detailed study information and a screening questionnaire. All participants provided signed electronic informed consent through the app. During a 28-day baseline phase, prescreened participants used the headache diary feature within the app to document headache attacks and medication intake. Furthermore, a physician conducted a video consultation to verify identity and migraine diagnosis, assess inclusion and exclusion criteria, and obtain final oral informed consent. The final study inclusion was only possible after the completion of the 28-day baseline headache and medication diary and the baseline questionnaire (Figure 1). After a 12-week intervention period, we evaluated the outcomes.

### Participant Recruitment and Eligibility

Recruitment for the trial took place during a 23-week period from March to December 2021 with a wide range of recruitment approaches having a focus on poster advertisements, online social media ads, and influencer campaigns. There was no face-to-face recruitment by research personnel. All participants received an incentive in the form of an online shopping voucher for €80 (approximately $90 USD), which was granted after completion of the study, and access to the full app version for 1 year.

Eligible participants were adults at least 18 years of age diagnosed with migraine (*International Statistical Classification of Diseases, Tenth Revision* code G43 with all subcodes, confirmed in video consultation by a study physician according to the *International Classification of Headache Disorders*, version 3 [*ICHD-3*],^17,18^ diagnosis criteria), having a disease duration of at least 1 year, and an onset of the disease before the age of 50 years. Participants had to document at least 3 migraine days throughout the 28-day baseline phase in the app, own a smartphone, and report smartphone literacy. Additionally, they were required to have sufficient proficiency in the German language and provide informed consent. Patients were not eligible if they fulfilled at least 1 of the following exclusion criteria: planned pregnancy or were pregnant and breastfeeding (due to associations between hormone status and migraine attacks); experienced medication overuse headache, as assessed by the physician and validated by app data during baseline (>15 days with medication use); used the study’s prescribable digital health app or a similar headache app for at least 1 month during the last 12 months; planned to start a new treatment for migraine within the next 4 months; participated simultaneously in another interventional study; and filled in the app headache diary fewer than 3 days per week during baseline.

### Randomization and Masking

Eligible participants were randomized after the baseline phase in a 1:1 ratio by block randomization with a fixed block length of 10 to either the intervention group or the control group. We used central randomization with a server-based randomization table implemented within the app. The randomization list was created by a statistician, who was otherwise not involved in the study, using the RANUNI random number generator of the SAS/STAT version 9.2 (SAS Institute Inc). Randomization was stratified based on the type of migraine (episodic or chronic). To ensure allocation concealment, neither the user nor the study staff with potential participant contact had access to the randomization list.

### Intervention

Patients in the intervention group were given access to the study’s full prescribable digital health app. The app was certified as a class I medical device based on the Medical Device Directive and fulfilled all German DiGA requirements for an app on prescription.^1^ M-sense Migräne (Newsenselab GmbH) included a headache-, trigger-, and medication-diary, a data report feature, and an active self-management feature, which offered nonpharmacological interventions and trigger management strategies (Table 1). Use patterns could vary and depended on individual needs (eg, serving as a headache diary or a comprehensive treatment program). The intervention operated exclusively through the app interface, without human involvement (examples of app screenshots are given in the eFigure in Supplement 2, and the theoretical foundation is provided in eMethods 1 in Supplement 2).

**Table 1.** Features of the Prescribable Digital Health App Used in the Study

App feature and module	Key functions and content
Diary	
Headache and medication diary	Standardized symptom and medication intake documentation; in-app algorithm to classify attacks according to *ICHD-3*^18^
Trigger diary	Selection of 12 trackable trigger factors (eg, sleep duration, water intake)
Data analysis and report	
Headache analysis	Descriptive analysis; visualization of headache attacks
Medication intake analysis	Visualization of intake patterns; automated warnings of medication overuse
Trigger analysis	Mean values for nonheadache days, headache days, and 2 days preceding a headache attack; menstrual cycle analysis for women
Data export	Monthly PDF report for physicians; includes automated attack classification and descriptive analysis
Active self-management	
Education module	33 Personalized knowledge lessons; rule-based interactive chatbot
Relaxation module	Audio for progressive muscle relaxation, autogenic training, breathing, and imagination
Training module	Instructions for endurance sports; training plan
Behavioral migraine management	Goal setting for lifestyle adjustments; reminders and feedback loops

Abbreviations: *ICHD-3*, *International Classification of Headache Disorders*, version 3; PDF, portable document format.

### Control App

The control app was a reduced app configuration of the study’s prescribable digital health app with only a headache and medication intake diary. Users could not view their headache attack classification or descriptive statistics about headache patterns. Headache entries could only be seen retrospectively for 1 week. The trigger diary and the Active self-management features were not accessible.

### Outcomes

The primary outcome was migraine days per month (defined as 28 days) after 12 weeks (assessed from weeks 9 to 12) based on the in-app headache diary. Following the final data collection, migraine attacks were classified according to *ICHD-3*^17^ (criteria B to D) by a physician blinded to the patient’s group allocation. A migraine day was defined as a calendar day on which a single or multiple migraine attacks were reported.

Prespecified secondary outcomes, assessed after 12 weeks, were headache days per month (defined as 28 days), days per month with moderate or severe headaches (lasting longer than 4 hours and rated at least 4 in intensity on an 11-point numeric rating scale ranging from 0, indicating no pain, to 10, indicating worst pain), days with acute headache medication including triptan use per month, days with triptan use per month (triptan as migraine-specific medication), days with acute headache medication use without triptans per month, responder rate (at least 30% reduction in migraine days per month), the 6-item Headache Impact Test (HIT-6) score, which ranges from 36 to 78, with lower values indicating better status,^19^ Headache Management Self-Efficacy Scale German Short Form (HMSE-G-SF) score, which ranges from 6 to 42, with higher values indicating better status,^20^ Headache-Attributed Lost Time in the previous 30 days (HALT-30) score, which ranges from 0 to 30, with lower values indicating better status,^21^ and a migraine-specific health literacy questionnaire (in-house–developed 3-item questionnaire assessing disease, trigger, and prophylaxis literacy), with each part ranging from 1 to 4, with lower values indicating better status. Safety was also assessed.

### Procedures

All study data were collected directly through the respective app. Alongside the diary, both the intervention and the control groups were provided with additional in-app questionnaires at baseline and after 12 weeks.

All participants received a daily notification with a headache question. If participants reported a headache, they were seamlessly redirected to log further details in the app diary. A response of no headache was also recorded. A day without a response to the daily headache question or a diary entry was considered as missing data.

For the safety assessment, data regarding suspected adverse reactions (SAR) and serious adverse events (SAE) were collected within the app at 4, 8, and 12 weeks. Throughout the trial, the study team (D.P. and C.M.W.) continually evaluated the SAE data for each patient to determine whether there was any relationship with the study intervention. Additionally, participants could reach out to the study team if they had any questions, need for technical support, or acute problems with the study or the app.

A follow-up study was conducted to measure the previously mentioned outcomes in the intervention group at the 24-week mark. The analysis of the follow-up study findings is pending.

### Statistical Analysis

All statistical analyses were performed in the statistical programming language R,^22^ version 4.0.2 (R Project for Statistical Computing), including double programming for the main analysis, from February to March 2022. Details of the analysis and the sample size calculation were prespecified and are provided in the statistical analysis plan (in Supplement 1). The threshold for statistical significance was *P* < .05 for the test of the primary hypothesis. Further statistical tests for the primary outcome, and all secondary analyses are to be interpreted as exploratory. All statistical tests were 2-sided. All analyses followed the intention-to-treat principle, and missing data were imputed (Supplement 1).

The primary outcome of the number of migraine days between weeks 9 and 12 was analyzed in the intention-to-treat population via an analysis of covariance (ANCOVA) model including randomized treatment group, stratification variable (episodic or chronic migraine), and baseline migraine days as independent variables, with corresponding adjusted means reported for each treatment group. Gender (diverse, female, and male), number of baseline migraine days, type of migraine, and disease duration were analyzed with separate ANCOVAs with interaction terms.

Secondary outcomes were analyzed with ANCOVA models. The treatment group, stratification variable, and the baseline value of the corresponding outcome were included as independent variables. Only the comparison of the responder rate (defined as achieving at least a 30% reduction in migraine days per month) was performed with logistic regression adjusting for the number of baseline migraine days and a stratification variable, and results were reported as odds ratios with 95% CIs.

Patients who did not answer the daily headache question (in-app push notification) at least 3 days per week for at least 4 weeks consecutively were considered as study dropouts. Patients in the intervention group who were not adherent to the intervention for at least 4 weeks consecutively were considered as intervention dropouts. Different methods to handle the missing data for these patients were prespecified and are described in eMethods 2 in Supplement 2. The numbers of study dropouts were compared with a Fisher exact test between treatment groups and are reported in eTable 4 in Supplement 2.

## Results

### Participants

**Figure 2.**
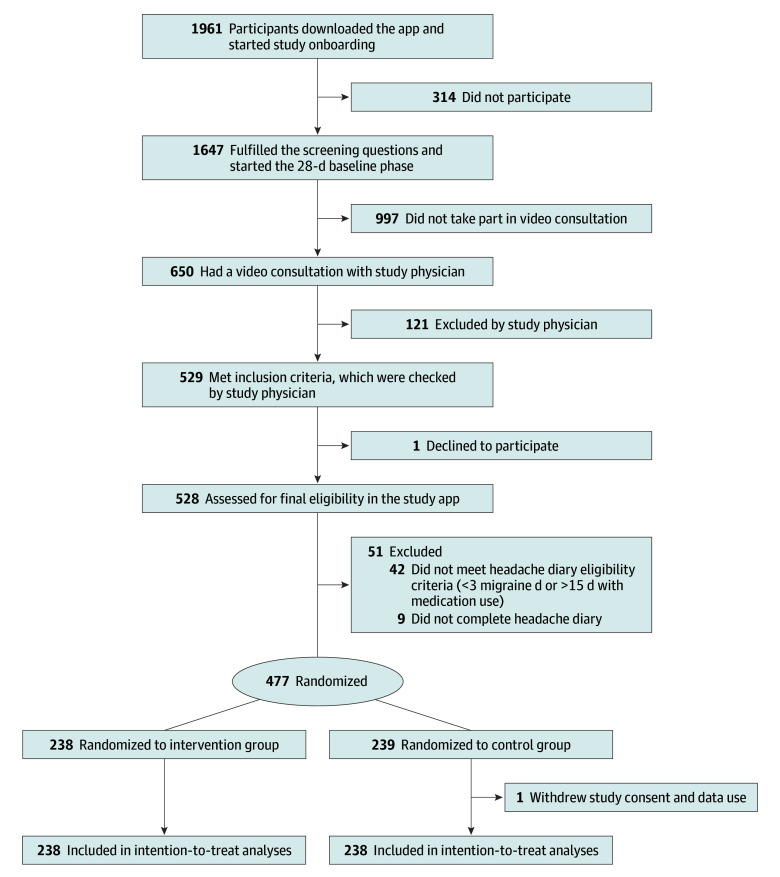
CONSORT Flowchart

Among 1961 interested participants who downloaded the app and started the study onboarding, 477 participants (24.3%) enrolled and were randomized. Details on the study flow and randomization are shown in Figure 2. One user from the control group withdrew participation and data use shortly after randomization, resulting in 238 participants in the control group.

Overall, most study participants were female (429 [90.1%]; 0 diverse and 45 [9.4%] male) and had a mean (SD) age of 35.2 (10.4) years (Table 2). Episodic migraine was reported by 225 participants (94.5%) in both the control and intervention groups, with an overall mean (SD) migraine history of 17.42 (10.49) years. During the 28-day baseline period, participants reported a high mean (SD) of 9.37 (4.00) headache days, including 6.48 (4.07) days with moderate or severe headaches. A mean (SD) of 7.75 (3.69) days was classified as migraine days. The mean (SD) HIT-6 score was 64.31 (3.67), and the mean (SD) HALT-30 score was 11.37 (7.59), indicating a high disease burden in the population. Baseline characteristics were comparable between both groups and are shown in Table 2.

**Table 2.** Participant Baseline Characteristics

Characteristic	Participants, No. (%)^a^	
	Intervention (n = 238)	Control (n = 238)
Age, mean (SD), y	35.6 (10.3)	34.8 (10.5)
Gender		
Diverse	0	0
Female	211 (88.7)	218 (91.6)
Male	27 (11.3)	18 (7.6)
Not answered	0	1 (0.4)
Missing	0	1 (0.4)
BMI, mean (SD)	25.21 (5.49)	25.01 (5.74)
Educational level^b^		
High school diploma with university readiness	92 (38.7)	95 (39.9)
High school diploma	46 (19.3)	37 (15.5)
Technical college degree	39 (16.4)	46 (19.3)
Vocational training	4 (1.7)	11 (4.6)
Other	57 (23.9)	48 (20.2)
Missing	0	1 (0.4)
Migraine history, first manifestation, mean (SD), y	17.38 (10.69)	17.46 (10.31)
Migraine history, first diagnosis, mean (SD), y	11.92 (8.97)	11.87 (9.15)
Migraine type		
Episodic	225 (94.5)	225 (94.5)
Chronic	13 (5.5)	13 (5.5)
Migraine d/mo, mean (SD)^c^	7.74 (3.70)	7.76 (3.69)
Headache d/mo, mean (SD)^c^	9.59 (4.05)	9.14 (3.93)
Moderate or severe headache d/mo, mean (SD)^c^	6.47 (4.22)	6.49 (3.91)
Migraine-specific quality of life, HIT-6 score, mean (SD)^d^	64.18 (3.53)	64.43 (3.81)
Headache attributed burden, HALT-30 score, mean (SD)^e^	10.43 (7.07)	12.32 (7.99)
Self-efficacy, HMSE-G-SF score, mean (SD)^f^	24.24 (6.89)	23.37 (6.83)
Migraine-specific health literacy score, mean (SD)^g^		
Prophylaxis	2.34 (0.68)	2.40 (0.70)
Trigger	2.35 (0.66)	2.39 (0.74)
Disease	2.35 (0.73)	2.42 (0.79)
Days per mo with acute headache medication use, mean (SD)^c^		
Total pain medication use (triptan and others)	5.13 (2.48)	4.84 (2.42)
Triptan use	3.97 (2.41)	4.05 (2.63)
Pain medication use (without triptan)	3.77 (2.25)	3.65 (2.14)

Abbreviations: BMI, body mass index (calculated as weight in kilograms divided by height in meters squared); HALT-30, Headache-Attributed Lost Time; HIT-6, 6-item Headache Impact Test; HMSE-G-SF, Headache Management Self-Efficacy Scale German Short Form.

^a^ Percentages may not sum to 100% due to missing data.

^b^ The German educational system is structured differently than that in many English-speaking countries. Approximate English counterparts are presented in order for Hochschulreife, Fachhochschulreife, Berufsfachschule, and Abgeschlossene Lehre, respectively.

^c^ Based on entries in the 28-day baseline diary.

^d^ HIT-6 scores range from 36 to 78, with lower values indicating better status.

^e^ HALT-30 scores range from 0 to 30, with lower values indicating better status.

^f^ HMSE-G-SF scores range from 6 to 42, with higher values indicating better status.

^g^ Scores range from 1 to 4, with lower values indicating better status.

### Primary Outcome

For the primary outcome (migraine days per month, defined as 28 days) after 12 weeks (assessed weeks 9 to 12) no significant differences were observed between the 2 groups (between-group difference [intervention minus control], 0.38 [95% CI, −0.32 to 1.08] days; *P* = .29). This result remained consistent when applying different multiple imputation methods in sensitivity analyses (eTables 1, 2, and 3 in Supplement 2). Both groups documented approximately 2 days less with migraine during weeks 9 to 12 compared with the 4-week baseline period (intervention group mean [SD], 7.74 [3.70] days during baseline to an adjusted mean of 5.79 [95% CI, 5.02-6.57] days during weeks 9 to 12; control group mean [SD], 7.76 [3.69] days during baseline to an adjusted mean of 5.41 [95% CI, 4.63-6.19] days during weeks 9 to 12). Evidence of a moderating treatment effect was found for none of the assessed potential moderators, including gender (male, female, and other, which included not answered or missing; interaction *P* = .40), baseline migraine days (low vs high based on median split baseline migraine days; interaction *P* = .68), type of migraine disease (chronic or episodic migraine; interaction *P* = .37), and duration of migraine disease (median split duration of migraine; interaction *P* = .65).

### Secondary Outcomes

No evidence for differences was found for the following secondary outcome measures: headache days per month, days per month with moderate or severe headaches, days with acute headache medication use per month, days with triptan use per month, days with acute headache medication use (without triptans) per month, responder rate, HIT-6 score (adjusted mean difference, −0.72 [95% CI, −1.78 to 0.34]; *P* = .17), HMSE-G-SF score (adjusted mean difference, 1.48 [95% CI, −0.14 to 3.10]; *P* = .07), and HALT-30 score (adjusted mean difference, −1.41 [95% CI, −6.78 to 3.96]; *P* = .52) after 12 weeks (*P* values are exploratory) (Table 3). Regarding migraine-specific health literacy, differences in scores between groups were observed in prophylaxis literacy (−0.14 [95% CI, −0.25 to −0.03]; *P* = .01) and disease literacy (−0.29 [95% CI, −0.39 to −0.19]; *P* < .001) in favor of the intervention group at week 12.

**Table 3.** Results From the Intention-to-Treat Analyses for Primary and Secondary Outcomes

Outcome	Adjusted mean (95% CI)			*P* value
	Intervention (n = 238)	Control (n = 238)	Difference	
No. of migraine d/mo during wk 9-12 (primary outcome)	5.79 (5.02 to 6.57)	5.41 (4.63 to 6.19)	0.38 (−0.32 to 1.08)	.29
No. of migraine d/mo				
Week 1-4	7.72 (7.01 to 8.43)	6.38 (5.67 to 7.10)	1.34 (0.76 to 1.91)	<.001
Week 5-8	6.76 (6.01 to 7.51)	5.98 (5.24 to 6.73)	0.78 (0.07 to 1.48)	.03
No. of headache d/mo				
Week 1-4	9.41 (8.61 to 10.20)	8.07 (7.30 to 8.85)	1.33 (0.70 to 1.97)	<.001
Week 5-8	8.16 (6.56 to 9.77)	7.47 (6.36 to 8.57)	0.70 (−0.94 to 2.33)	.34
Week 9-12	7.56 (5.74 to 9.38)	7.08 (5.80 to 8.36)	0.48 (−1.41 to 2.37)	.56
Responder rate after 12 wk^a^	0.49 (0.37 to 0.61)	0.57 (0.45 to 0.69)	0.72 (0.44 to 1.17)^b^	.19
No. of moderate and severe headache d/mo				
Week 1-4	6.75 (5.75 to 7.75)	5.53 (4.72 to 6.35)	1.22 (0.47 to 1.96)	.002
Week 5-8	6.53 (5.51 to 7.55)	5.67 (4.45 to 6.89)	0.86 (0.18 to 1.54)	.01
Week 9-12	6.22 (4.51 to 7.94)	4.99 (3.77 to 6.21)	1.23 (−0.58 to 3.04)	.15
No. of d/mo with acute headache medication use				
Week 1-4	4.43 (3.89 to 4.97)	3.51 (3.11 to 3.90)	0.92 (0.36 to 1.48)	.002
Week 5-8	3.92 (3.52 to 4.33)	3.51 (3.05 to 3.97)	0.41 (−0.25 to 1.07)	.20
Week 9-12	3.65 (3.06 to 4.24)	3.23 (2.77 to 3.69)	0.42 (−0.47 to 1.32)	.31
No. of d/mo with triptan use				
Week 1-4	2.34 (1.43 to 3.24)	1.88 (1.18 to 2.58)	0.45 (−0.18 to 1.08)	.19
Week 5-8	2.15 (1.41 to 2.89)	1.81 (0.95 to 2.68)	0.34 (−0.60 to 1.28)	.42
Week 9-12	1.90 (1.24 to 2.56)	1.55 (0.89 to 2.21)	0.35 (−0.11 to 0.80)	.13
No. of d/mo with acute headache medication use (without triptans)				
Week 1-4	2.86 (2.45 to 3.28)	2.18 (1.64 to 2.71)	0.69 (0.24 to 1.14)	.003
Week 5-8	2.85 (2.45 to 3.24)	2.39 (1.81 to 2.98)	0.45 (−0.01 to 0.91)	.05
Week 9-12	2.58 (2.22 to 2.95)	2.25 (1.67 to 2.84)	0.33 (−0.30 to 0.96)	.27
HIT-6 score after 12 wk^c^	63.53 (62.57 to 64.48)	64.25 (63.64 to 64.85)	−0.72 (−1.78 to 0.34)	.17
HMSE-G-SF score after 12 wk^d^	23.84 (21.58 to 26.10)	22.36 (19.59 to 25.14)	1.48 (−0.14 to 3.10)	.07
HALT-30 score after 12 wk^e^	10.66 (7.55 to 13.78)	12.07 (10.66 to 13.48)	−1.41 (−6.78 to 3.96)	.52
Migraine-specific health literacy score after 12 wk^f^				
Prophylaxis	2.20 (2.12 to 2.28)	2.34 (2.25 to 2.43)	−0.14 (−0.25 to −0.03)	.01
Disease	2.05 (1.98 to 2.13)	2.34 (2.27 to 2.41)	−0.29 (−0.39 to −0.19)	<.001
Trigger	2.21 (1.90 to 2.52)	2.26 (2.15 to 2.38)	−0.05 (−0.61 to 0.51)	.83

Abbreviations: HALT-30, Headache-Attributed Lost Time; HIT-6, 6-item Headache Impact Test; HMSE-G-SF, Headache Management Self-Efficacy Scale German Short Form.

^a^ Proportion.

^b^ Odds ratio.

^c^ HIT-6 scores range from 36 to 78, with lower values indicating better status.

^d^ HMSE-G-SF scores range from 6 to 42, with higher values indicating better status.

^e^ HALT-30 scores range from 0 to 30, with lower values indicating better status.

^f^ Scores range from 1 to 4, with lower values indicating better status.

### Safety

SAEs were reported by 3 participants (2 intervention, 1 control). During the blinded event assessment, only 1 of these events was plausibly related to the study, but this was considered unlikely and was reported by a participant in the control group (hospitalization due to unspecific symptoms). In our study, an SAR was defined as an event that the app user directly associated (self-reported) with the app use. In the intervention group, 5 participants reported a total of 6 SARs, while in the control group, 2 participants reported a total of 2 SARs. The SARs included 4 reports of increased awareness toward migraine attacks and headache pain due to the daily reminders and diary feature. One participant reported the beginning of a headache attack associated with the use of a massage ball and possible irritation of nerves and muscles. Another participant reported increased awareness and frustration about the disease burden due to app use. No participant withdrew from the study due to safety concerns.

## Discussion

In this RCT, a prescribable digital health app was not superior in reducing the number of migraine days after 12 weeks compared with a control app, which included only a basic headache diary. Most secondary outcome measures also did not show group differences. Overall, the intervention was safe.

Drawing comparisons with existing literature proved to be difficult due to a scarcity of research focusing on digital interventions for headache management. In a systematic review of RCTs, Noser et al^23^ observed that the field of testing the efficacy of digital interventions for headache management is still emerging, with most investigations being preliminary pilot studies involving small, homogenous groups of participants. More than half of the studies included in the review (7 of 13) used interactive websites and showed encouraging results. However, most web-based platforms were supplemented by some interactions with therapists or were an adjunct to in-person therapy. Only 3 studies of headache management apps were identified, and none of them showed significant differences compared with a waitlist control^24^ or active control groups that received either a limited app version^25^ or a sham app.^26^ Similarly, a recent meta-analysis by Huang et al^27^ found that, in the context of digital cognitive behavior therapy for headache, only web-based interventions have been investigated so far, with no studies specifically examining headache apps.

To the best of our knowledge, our trial represents the first RCT that could be completed within the DiGA fast-track trial phase and is the first decentralized, fully remote RCT to evaluate a migraine app. Despite a COVID-19 pandemic lockdown throughout the trial phase, we successfully achieved the necessary sample size in a relatively short recruitment period by using modern advertising techniques, especially social media ads and influencer campaigns. Furthermore, the advanced approach to address dropouts and the fully reproducible statistical analysis adhered to a prespecified analysis plan with several sensitivity analyses for the primary outcome. The programming for the primary outcome analysis was performed independently by 2 statisticians (including P.H.) and cross-checked, to account for the complexity of condensing the outcome data.

### Limitations

This study had limitations. Both the intervention and the control group used an in-app diary to document headache attacks to avoid substantial differences between groups, even though this approach did not allow for blinding of participants to group assignments. However, it is essential to acknowledge that tracking itself is already an integral component of the app under investigation. A systematic review^28^ highlighted the widespread use of electronic headache diaries among patients with headaches and the potential for multiple effects. We may have underestimated the therapeutic effect of using a diary in a modern digital format, as the control condition did not represent a true no-treatment group.

While designing the control app, we intentionally chose to allow access only to the basic headache and medication diary. This may have resulted in differences in documenting attacks and medication intakes in both groups. The intervention group with access to the full version of the app had more entry points to the diary. Additionally, the intervention group was educated about the importance of detailed tracking, rewarded for daily documentation, received immediate feedback on their entries (eg, a warning regarding medication overuse), and had access to an analysis function. This could have created a greater incentive in the intervention group to document headache attacks and medication intake more conscientiously.

As part of the German DiGA process, all essential tasks of the clinical trial had to be completed within a 12-month time frame. Consequently, the intervention period was limited to 12 weeks, which may have been too short for participants to adequately adopt trigger management and nonpharmacological interventions provided in the app and to detect meaningful changes in migraine frequency.

An additional limitation of our study concerns the potential for selection bias and reduced external validity, particularly due to the recruitment of a patient subgroup with migraine having relatively high baseline disease severity.^29,30^ This subgroup exhibited more frequent attacks and higher disease burden, as reflected in their high HIT-6 and HALT-30 scores. As a result, our sample may not accurately represent the broader migraine population, particularly patients with milder or more episodic forms of the condition. Furthermore, participants who voluntarily engage in remote, app-based trials may differ in meaningful ways from the general population. For example, they may be more proactive about managing their health, more comfortable with technology, or more motivated to seek alternative treatment options.

## Conclusions

In this RCT involving patients with migraine, there was no evidence for a superiority of the prescribable digital health app over a control app providing basic documentation features in reducing the number of migraine days across a 12-week period. However, our study highlighted the feasibility and rapid recruitment capabilities in digital health research along with the challenges of conducting a fully remote trial within the established German DiGA fast-track process. This study underscores the critical need for RCTs as a cornerstone of a robust evaluation framework, ensuring both treatment efficacy and patient safety in an expanding digital therapeutics market.

## Notes

Supplement 1.
Trial Protocol and Statistical Analysis Plan

Supplement 2. **eTable 1.** Results from sensitivity analyses with multiple imputation **eTable 2.** Results from sensitivity analyses with complete case analysis (FAS analysis) **eTable 3.** Results of sensitivity analysis with ANCOVA **eTable 4.** Dropout analysis **eFigure.** Screenshots of the “M-sense Migräne” app **eMethods 1.** Theoretical basis of applied behavior change theories and techniques in the app **eMethods 2.** Handling of dropouts and missing data imputation

Supplement 3.
Data Sharing Statement

## References

[1] Digital Health Applications. Federal Institute for Drugs and Medical Devices. Accessed December 20, 2024, https://www.bfarm.de/EN/Medical-devices/Tasks/DiGA-and-DiPA/Digital-Health-Applications/_node.html

[2] Mantovani A, Leopaldi C, Nighswander CM, Di Bidino R. Access and reimbursement pathways for digital health solutions and *in vitro* diagnostic devices: current scenario and challenges. Front Med Technol. 2023;5:1101476. doi:10.3389/fmedt.2023.1101476 36891483 PMC9986593

[3] Stewart WF, Shechter A, Rasmussen BK. Migraine prevalence. a review of population-based studies. Neurology. 1994;44(6)(suppl 4):S17-S23. 8008222

[4] Becker WJ, Findlay T, Moga C, Scott NA, Harstall C, Taenzer P. Guideline for primary care management of headache in adults. Can Fam Physician. 2015;61(8):670-679. 26273080 PMC4541429

[5] Kropp P, Meyer B, Dresler T, . Relaxation techniques and behavioural therapy for the treatment of migraine: guidelines from the German Migraine and Headache Society. Entspannungsverfahren und verhaltenstherapeutische Interventionen zur Behandlung der Migrane. Schmerz. 2017;31:433-447. doi:10.1007/s00482-017-0214-1 28364171

[6] Headaches in over 12s: diagnosis and management. National Institute for Health and Care Excellence. Updated December 17, 2021. Accessed August 18, 2023. https://www.nice.org.uk/guidance/cg150/chapter/Recommendations#management-2 32017486

[7] Hundert AS, Huguet A, McGrath PJ, Stinson JN, Wheaton M. Commercially available mobile phone headache diary apps: a systematic review. JMIR Mhealth Uhealth. 2014;2(3):e36. doi:10.2196/mhealth.3452 25138438 PMC4147710

[8] Bromberg J, Wood ME, Black RA, Surette DA, Zacharoff KL, Chiauzzi EJ. A randomized trial of a web-based intervention to improve migraine self-management and coping. Headache. 2012;52(2):244-261. doi:10.1111/j.1526-4610.2011.02031.x 22413151 PMC3305283

[9] Flynn N. Effect of an online hypnosis intervention in reducing migraine symptoms: a randomized controlled trial. Int J Clin Exp Hypn. 2019;67(3):313-335. doi:10.1080/00207144.2019.1612674 31251706

[10] T Minen M, Adhikari S, K Seng E, . Smartphone-based migraine behavioral therapy: a single-arm study with assessment of mental health predictors. NPJ Digit Med. 2019;2:46. doi:10.1038/s41746-019-0116-y 31304392 PMC6550263

[11] Sorbi MJ, Balk Y, Kleiboer AM, Couturier EG. Follow-up over 20 months confirms gains of online behavioural training in frequent episodic migraine. Cephalalgia. 2017;37(3):236-250. doi:10.1177/0333102416657145 27558500

[12] Neeb L, Ruscheweyh R, Dresler T. Digitalization in headache therapy. Digitalisierung in der Kopfschmerzbehandlung. Schmerz. 2020;34:495-502. doi:10.1007/s00482-020-00508-3 PMC7529087 33006064

[13] Raffaelli B, Mecklenburg J, Overeem LH, . Determining the evolution of headache among regular users of a daily electronic diary via a smartphone app: observational study. JMIR Mhealth Uhealth. 2021;9(7):e26401. doi:10.2196/26401 34255716 PMC8295831

[14] Diener HC, Tassorelli C, Dodick DW, ; International Headache Society Clinical Trials Committee. Guidelines of the International Headache Society for controlled trials of preventive treatment of migraine attacks in episodic migraine in adults. Cephalalgia. 2020;40(10):1026-1044. doi:10.1177/0333102420941839 32722936

[15] Tassorelli C, Diener HC, Dodick DW, ; International Headache Society Clinical Trials Standing Committee. Guidelines of the International Headache Society for controlled trials of preventive treatment of chronic migraine in adults. Cephalalgia. 2018;38(5):815-832. doi:10.1177/0333102418758283 29504482

[16] Schulz KF, Altman DG, Moher D; CONSORT Group. CONSORT 2010 statement: updated guidelines for reporting parallel group randomised trials. BMJ. 2010;340:c332. doi:10.1136/bmj.c332 20332509 PMC2844940

[17] Headache Classification Committee of the International Headache Society (IHS). The International Classification of Headache Disorders, 3rd edition. *Cephalalgia*. 2018;38(1):1-211. doi:10.1177/0333102417738202 29368949

[18] Roesch A, Dahlem MA, Neeb L, Kurth T. Validation of an algorithm for automated classification of migraine and tension-type headache attacks in an electronic headache diary. J Headache Pain. 2020;21(1):75. doi:10.1186/s10194-020-01139-w 32532222 PMC7291668

[19] Kosinski M, Bayliss MS, Bjorner JB, . A six-item short-form survey for measuring headache impact: the HIT-6. Qual Life Res. 2003;12(8):963-974. doi:10.1023/A:1026119331193 14651415

[20] Graef JE, Rief W, French DJ, Nilges P, Nestoriuc Y. German language adaptation of the Headache Management Self-Efficacy Scale (HMSE-G) and development of a new short form (HMSE-G-SF). Headache. 2015;55(7):958-972. doi:10.1111/head.12564 25904007

[21] Steiner TJ, Lipton RB; Lifting The Burden: The Global Campaign against Headache. The Headache-Attributed Lost Time (HALT) Indices: measures of burden for clinical management and population-based research. J Headache Pain. 2018;19(1):12. doi:10.1186/s10194-018-0837-3 29396646 PMC5796955

[22] The R Project for Statistical Computing. R Core Team. Accessed April 20, 2025. https://www.R-project.org/

[23] Noser AE, Gibler RC, Ramsey RR, Wells RE, Seng EK, Hommel KA. Digital headache self-management interventions for patients with a primary headache disorder: a systematic review of randomized controlled trials. Headache. 2022;62(9):1105-1119. doi:10.1111/head.14392 36286601 PMC10336649

[24] Minen MT, Corner S, Berk T, . Heartrate variability biofeedback for migraine using a smartphone application and sensor: a randomized controlled trial. Gen Hosp Psychiatry. 2021;69:41-49. doi:10.1016/j.genhosppsych.2020.12.008 33516964 PMC8721520

[25] Minen MT, Adhikari S, Padikkala J, . Smartphone-delivered progressive muscle relaxation for the treatment of migraine in primary care: a randomized controlled trial. Headache. 2020;60(10):2232-2246. doi:10.1111/head.14010 33200413 PMC8721526

[26] Stubberud A, Linde M, Brenner E, . Self-administered biofeedback treatment app for pediatric migraine: a randomized pilot study. Brain Behav. 2021;11(2):e01974. doi:10.1002/brb3.1974

[27] Huang YB, Lin L, Li XY, Chen BZ, Yuan L, Zheng H. An indirect treatment comparison meta-analysis of digital versus face-to-face cognitive behavior therapy for headache. NPJ Digit Med. 2024;7(1):262. doi:10.1038/s41746-024-01264-9 39343978 PMC11439962

[28] van de Graaf DL, Schoonman GG, Habibović M, Pauws SC. Towards eHealth to support the health journey of headache patients: a scoping review. J Neurol. 2021;268(10):3646-3665. doi:10.1007/s00415-020-09981-3 32529582 PMC8463346

[29] Cohen F, Brooks CV, Sun D, . Prevalence and burden of migraine in the United States: A systematic review. Headache. 2024;64(5):516-532. doi:10.1111/head.14709 38700185

[30] Porst M, Wengler A, Leddin J, . Migraine and tension-type headache in Germany: prevalence and disease severity from the BURDEN 2020 Burden of Disease Study. J Health Monit. 2020;5(suppl 6):2-24. doi:10.25646/6990.2 35146296 PMC8734075

