# Supplementary material for: A Prescribed Digital Health App and Number of Migraine Days: A Randomized Clinical Trial
Source: JAMA Netw Open. 2025 Jul 1;8(7):e2517708. doi: 10.1001/jamanetworkopen.2025.17708 (PMC12215570; doi:10.1001/jamanetworkopen.2025.17708)
Supplement: Supplement 1. — Trial Protocol and Statistical Analysis Plan [file jamanetwopen-e2517708-s001.pdf]

# EVALUATION CONCEPT

# Effectiveness of the M-sense Migräne app – a randomized controlled study - EMMA Study

Version number 2.1

Date: December 09, 2020

# EVALUATION CONCEPT REVISION HISTORY

| Previous Version | Updated Version | Sections changed | Description and reason for change | Date of change |
|---|---|---|---|---|
| | 0.0 | | Creation of document | 20.05.2020 |
| 0.0 | 1.0 | | First final version | 26.08.2020 |
| 1.0 | 1.1 | Synopsis, Table 1, 6.3, 11.2, 11.4, 11.9, 13.2 | First revised version | 22.11.2020 |
| 1.1 | 2.0 | Synopsis, 3, 4.1, 4.2, 6.2, 6.3, Table 2, 11.1, 11.3, 11.6, 11.7, 13 | Second revised version | 05.12.2020 |
| 2.0 | 2.1 | 4.2, 6.2, 11.6 | Classification of migraine days changed | 09.12.2020 |

# SYNOPSIS

| | |
|---|---|
| **Study title** | Effectiveness of the **M-sense Migräne a**pp – a randomized controlled study - EMMA Study |
| **Type of project** | Clinical study on the effectiveness of the M-sense app for patients with migraine |
| **Study phase** | Phase IV, study according to § 23 b MPG (Ausnahme der klin. Prüfung) |
| **Sponsor** | Newsenselab GmbH (Contact: Julian Gansen)<br>Blücherstraße 22<br>10961 Berlin, German<br>Phone: +49 173 7070 903<br>e-mail: gansen@newsenselab.com |
| **Principle investigator** | Charité – Universitätsmedizin Berlin<br>Institut für Sozialmedizin, Epidemiologie und Gesundheitsökonomie<br>Luisenstr. 57<br>Prof. Dr. Claudia M. Witt<br>10117 Berlin<br>Tel: +49 30 450 529 132<br>Fax: +49 30 450 529 917 |
| **Study aim** | The aim is to evaluate the effectiveness of the M-sense app in reducing migraine days in patients with migraine. |
| **Study rationale** | The M-sense Migräne app by Newsenselab GmbH includes numerous evidence-based features to improve symptoms and quality of life of migraine patients. With this app patients can document their headache attacks and medication intake in an electronic diary to enable monitoring of therapy and identification of migraine triggers. An integrated therapy module supports patients in the implementation of relaxation techniques and endurance sports, and trains them in individual behavior therapy approaches. Data can be exchanged with the treating physician.<br>This study aims to evaluate the M-sense Migräne app eligibility for inclusion in the DiGA directory – based on the Digital Healthcare Act (DVG) and Digital Health Applications Ordinance (DiGAV). |
| **Study objectives** | The aim is to evaluate the effectiveness of the M-sense Migräne app in reducing migraine days in episodic migraine and chronic migraine patients, compared to patients in a waiting list group who have no access to the app intervention.<br>Secondary objectives are the evaluation of headaches, migraine-specific |

| | |
|---|---|
| | health-related quality of life, safety, adherence, migraine-specific health literacy, and headache management self-efficacy of M-sense users. |
| **Study design** | This is a two-armed, open-label, parallel-group, randomized, controlled study. Patients will be randomly allocated to one of the two groups in a 1:1 ratio to gain access either to the full M-sense Migräne app or the Control study app with only data input features. For motivational reasons the control group participants will gain access to the full M-sense Migräne app after 12 weeks. This additional data and the 24-weeks follow-up in the intervention group will not be part of the DiGA-study. |
| **Interventions and controls** | Intervention group: M-sense Migräne app for 12 weeks.<br>Control group: Control study app that only includes data input features (as a diary visible for one week without attack classification) and questionnaires. There will be no app interventions and no data will be feedbacked to the patients. |
| **Investigational device** | *The M-sense Migräne app* includes a comprehensive mobile diary for tracking headache attacks, medication intake and trigger factors, as well as nonpharmacological interventions for headache prevention and acute pain therapy. |
| **Total number of patients** | Randomized population: n = 346 (n= 173 patients per group)<br>Analysis population (intention-to-treat, imputation of missing data): n = 346 |
| **Study population** | Adult patients with migraine (ICD-10 G43 = episodic and chronic migraine), who have a smartphone and are interested to use an app as non-pharmacological intervention. |
| **Inclusion criteria** | • Adults ≥ 18 years with migraine (ICD-10 G43) diagnosed by a physician<br>• Disease duration of at least 1 year (patient reported)<br>• At least 3 migraine days in the previous 28 days<br>• Disease onset before age 50 y<br>• Migraine screening questions positively answered<br>• Smartphone ownership and smartphone literacy<br>• Sufficient knowledge of German<br>• Informed consent |
| **Exclusion criteria** | • Planned pregnancy, pregnant and breastfeeding women (hormone status has an impact on the headache and migraine days)<br>• Medication overuse headache (assessed by physician question, app data during baseline > 15 days with medication)<br>• Headache app / M-sense or similar app use of at least a month during the last 12 months<br>• Planned start of a new treatment for migraine within the next 4 months<br>• Simultaneous participation in another interventional study<br>• Users who have filled in the diary <3 days/week during baseline |
| **Endpoints** | Primary endpoint: migraine days per month (defined as 28 days) after 12 weeks (week 9 to 12)<br>Secondary endpoints<br>• Headache days per month (defined as 28 days)<br>• Migraine specific quality of life HIT-6, (6 items, last 4 weeks)<br>• Headache management self-efficacy HMSE-G-SF (6 items, currently)<br>• Disability / Headache attributed lost time (HALT-30, 5 items, last 4 weeks)<br>• Migraine specific health literacy<br>• Drug consumption for acute treatment (in-app data)<br>Further measurements<br>• Adherence to intervention (in app data, plus Item on intervention implementation without app use)<br>• Safety assessment |

| | |
|---|---|
| | • Concomitant non-pharmacological interventions |
| **Safety** | Information about suspected adverse reactions (SAR) and serious adverse events (SAE) will be collected at 4, 8 and 12 weeks. Data will be evaluated and assessed continuously throughout the study by the study investigators for each patient to determine whether there is any relationship to the study interventions. |
| **Study procedures** | The pre-screening of potential study participants will be carried out by a web page. In case of eligibility a code for usage of the study app is offered.<br>In app informed consent on privacy aspects will be obtained. An anonymous 28 days baseline assessment will follow, which assesses the eligibility for the study. Close to the end of the baseline phase in a video consultation with a physician the migraine diagnosis will be confirmed, the main study eligibility will be checked and the informed consent will be obtained. Eligible patients receive a code from the physician. After participants complete the baseline phase eligibility criteria will be checked within the app (migraine days ≥3, filling in of the diary, no medication overuse). Eligible participants will then be asked within the app to enter the code received by the physician, participants will then be randomized within the app. They will participate in the DiGA study for 12 weeks according to treatment allocation. After the end of the DiGA study both groups will be able to use the app for further 12 weeks in a follow-up study. |
| **Time points of data assessment** | Data of all endpoints are documented within the apps. For symptoms and medication intake daily, for other secondary outcomes at baseline and after 12 weeks (+ 12 weeks as follow-up in addition to the DiGA-study), for safety additionally after 4 and 8 weeks. |
| **Discontinuation criteria for the entire study** | The study will be discontinued if less than 10% of the anticipated number of participants are recruited within the recruitment time. |
| **Risk-benefit analysis** | This study includes non-pharmacological evidence-based interventions for a chronic disease and is not expected to involve relevant risks or burdens for the participants. Each participant is informed about the applied interventions as well as about possible risks regarding privacy and data safety prior study participation. |
| **Statistical analysis** | The primary effectiveness analysis for the primary endpoint (mean of the migraine days week 9-12) will be conducted using an analysis of covariance (ANCOVA) with the treatment group as fixed factor and baseline migraine days as covariate. From this model, estimated means and 95 % confidence intervals will be obtained. The analysis of the primary endpoint will be based on the intention-to- treat (ITT) population, which consists of all randomized patients. Missing data will be imputed by using adequate multivariate imputation methods. |
| **Study schedule** | Baseline First-Participant-In: January 1, 2021<br>Study First-Participant-In: January 29, 2021<br>Baseline Last-Participant-Out: June 30, 2021<br>Study Last-Participant-Out: Sep 30, 2021<br>Data Analysis and report to BfArM: Dec 15, 2021 |

This evaluation concept is substituted by the Statistical Analysis Plan (SAP) for the digital health application (DiGA) study: 'Effectiveness of the M-sense Migräne app – a randomized controlled study - EMMA Study', that provides more in depths information on outcomes and their rational and analyses methods (version 2.1 from December 05, 2020).

# SIGNATURES

The signatories confirm that they have read this study protocol thoroughly and that they agree that it contains all essential information for the implementation of the study.

## Sponsor

[redacted]

9.12.2020
Date

Markus Dahlem

## Principal investigator

[redacted]

9.12.2020
Date

Claudia M. Witt, MD [redacted]

## Co-Principal investigator

[redacted]

9.12.2020
Date

Daniel Pach, MD

## Data manager

[redacted]

9.12.2020
Date

Katja Icke [redacted]

# TABLE OF CONTENTS

1 Team of investigators and investigation administrative structure ... 8
1.1 Sponsor ... 8
1.2 Study office and investigators ... 8
2 Introduction ... 9
3 Objectives ... 10
4 Investigation and procedures ... 11
4.1 Overall study design ... 11
4.2 Justification of study design and approach ... 11
5 Participants and group allocation ... 12
5.1 Recruitment ... 12
5.2 Inclusion and exclusion criteria ... 12
5.3 Verification of diagnosis ... 13
5.4 Assignment to study groups ... 13
5.5 Withdrawal of study participants from treatment or assessment ... 13
6 Outcomes ... 14
6.1 Summary of assessments ... 14
6.2 Primary outcome ... 14
6.3 Secondary outcomes ... 14
6.4 Further measurements ... 15
6.5 Mapping of outcomes to DVG categories ... 16
7 Intervention ... 16
7.1 Intervention group ... 16
7.2 Control group ... 18
7.3 Concomitant treatments ... 19
8 Safety ... 19
8.1 Risk-benefit assessment ... 19
9 Assessment of safety ... 19
10 Study Procedures ... 21
10.1 Pre-screening and pre-study screening log ... 21
10.2 Baseline and informed consent ... 21
10.3 Randomization ... 21
10.4 Procedures in case of pregnancy ... 22
10.5 Discontinuation of the study ... 22
11 Statistical methods and determination of sample size ... 22
11.1 Sample size calculations ... 22
11.2 Analysis plan ... 22
11.3 Primary comparison and hypotheses ... 22
11.4 Analysis populations ... 23
11.5 Participants demographics and baseline characteristics ... 23
11.6 Primary outcome definition ... 23
11.7 Analysis of the primary outcome ... 23
11.8 Secondary outcomes ... 23
11.9 Further analyses ... 24
11.10 Analyses of safety outcomes ... 24
11.11 Interim analyses ... 24
12 Quality control and quality assurance ... 24
12.1 Data Management ... 24
12.2 Data monitoring ... 24
13 Ethics and regulations ... 25
13.1 Insurance ... 25

13.2 Study registration ..... 26
14 Publication ..... 26
15 Study schedule ..... 26
16 Reference list ..... 27

# 1 TEAM OF INVESTIGATORS AND INVESTIGATION ADMINISTRATIVE STRUCTURE

## 1.1 Sponsor

Newsenselab GmbH (Contact: Julian Gansen)
Blücherstraße 22
10961 Berlin, German
Phone: +49 30-95999242
e-mail: gansen@newsenselab.com

## 1.2 Study office and investigators

**Principal investigator**
Professorin Claudia M. Witt, MD, MBA
Institute for Social Medicine, Epidemiology and Health Economics
Charité - Universitätsmedizin Berlin, Luisenstr. 57
10117 Berlin, Germany
Phone: +49-30-450529132
Fax: +49-30-450529917
e-mail: claudia.witt@charite.de

**Co-Principal investigator**
Dr. med. Daniel Pach
Institute for Social Medicine, Epidemiology and Health Economics
Charité Universitätsmedizin Berlin, Luisenstr. 57
10117 Berlin, Germany
Phone: +49-30-450529068
Fax: +49-30-450529917
e-mail: daniel.pach@charite.de

**Data management**
Katja Icke
Institute for Social Medicine, Epidemiology and Health Economics
Charité - Universitätsmedizin Berlin, Luisenstr. 57
10117 Berlin, Germany
Phone: +49-30-450529 076
Fax: +49-30-450 529917
e-mail: katja.icke@charite.de

# 2 INTRODUCTION

Migraine is a common disorder with a prevalence of 12 % in the general population, and is more frequent in women (18%) than in men (6%).[1] Symptom frequency, severity, and impact on patients' quality of life varies.

A treatment plan should consider not only the patient's diagnosis, symptoms, and co-existent or comorbid conditions, but also the patient's expectations, needs, and goals.[2 3] In order to improve the quality of life, prevent permanent disability and chronicity, preventive therapy (prophylaxis) is often necessary. Prophylactic migraine therapy includes drug and non-pharmacological treatments and a reduction of monthly migraine days can be achieved.[3-5] A common problem of drug therapy are insufficient effects and/or intolerable side effects.[6] Non-pharmacological therapies include regular aerobic endurance sports, relaxation techniques and cognitive-behavioral therapy (CBT) methods used in psychological pain therapy (pain management, trigger identification and coping[7]).[8] Access to on-site psychological therapy, especially CBT, is limited by the number of psychotherapists specialising in pain disorders, both in urban and even more so in rural areas.

Digital health applications (or apps) allow scalability, efficiency, and behavior change.[9] Michie et al. defined the smallest, observable, replicable intervention components with the potential to bring change in behavior as behavior change techniques (BCTs).[10 11] BCTs could also be "a systematic procedure included as an active component of an intervention designed to change behavior"[12]. For example, app features and functions can be designed based on different BCTs to improve user engagement[13]: "prompts/cues" could be implemented as an app notification to remind users to fill in questionnaires and "feedback on behavior" could potentially maintain the user's motivation by providing instant feedback. Besides, "goal-setting" and "self-monitoring" are also commonly implemented BCTs in smartphone apps. Digital health applications for headache care can offer new options such as an electronic headache diary which offer more features than the established paper-pencil based headache diaries, e.g. by automatic analysis of potential trigger factors or headache attack classification. In addition to a documentation function in the form of a headache and medication intake diary, apps can also contribute to a better understanding of the headache disorder through education and provide electronically based guidance for non-pharmacological interventions, such as evidence-based CBT exercises. This app data can provide healthcare professionals with information relevant for optimised and effective treatment. However, the current commercially available headache apps have not been evaluated for effectiveness, or their content is not evidence-based.[14]

Since 2020, digital medical devices can be prescribed to patients by physicians and psychotherapists within the framework of the DiGA – Digital Healthcare Act (Digitale-Versorgung-Gesetz) in Germany.[15 16] Digital medical devices such as smartphone-based programmes can thus be made reimbursable by statutory health insurances, and hence made broadly available to the public. Since many

smartphone-based therapies have yet to be evaluated for effectiveness, the DiGA provides the framework to gather this evidence within 12 months.

The smartphone app M-sense Migräne[17] (www.m-sense.de) aims to enable migraine patients to track headache attacks, symptoms, medication intake and trigger factors, as well as to have access to evidence-based and evidence-informed treatment options within a digital environment. Due to the individual data collected by the e-diary (electronical diary), education on disease- and trigger management, relaxation exercises and instructions for endurance sports can automatically be tailored to the app user and his/her specific needs. However, to be reimbursed by statutory health insurance companies in Germany the app's effectiveness has to be shown in a study. To achieve this aim a post-market clinical investigation according to Digitale-Versorgung-Gesetz (DVG) will be prepared.

# 3 OBJECTIVES

The aim of this study is to evaluate the effectiveness of the M-sense Migräne app in reducing migraine days in migraine patients (ICD-10 G43 = episodic and chronic migraine) compared to patients in a waiting list group who have no access to the app intervention.

As secondary objectives the study evaluates headache days, migraine-specific quality of life, disability/ headache attributed lost time, migraine-specific health literacy, and headache management self-efficacy. Of further interest are the evaluation of safety, adherence, and drug consumption for acute treatment, concomitant non-pharmacological interventions.

# 4 INVESTIGATION AND PROCEDURES

## 4.1 Overall study design

This is a two-armed, open-label, parallel-group, randomized, controlled trial. Potential study participants will be invited to participate in a 28-days baseline phase in which data on headache days and migraine attacks will be collected. This data will be used for the eligibility check. Furthermore, a physician (via video consultation) will inform about the study, validate the patient-reported migraine diagnoses and obtain the informed consent from study participants. After final informed consent and verification of the patient's eligibility for the study in the video consultation, patients will be randomly allocated to one of the two groups in a 1:1 ratio to gain access either to the full M-sense Migräne app or the control study app with only data input features (only as a diary visible for one week without attack classification with daily headache question and questionnaires, no app interventions and no feedback on data or behavior). The primary outcome will be the migraine days per month (defined as 28 days in weeks 9 to 12).

The DiGA-study will be completed after 12 weeks follow-up per study participant.

Additional data will be collected afterwards. Then both groups receive the full M-sense Migräne app and use the intervention for 12 weeks (see Figure 1). This will allow to gather data over 24 weeks in the intervention group and increase the motivation in the control group to participate in the study and to provide data over 12 weeks without access to the intervention.

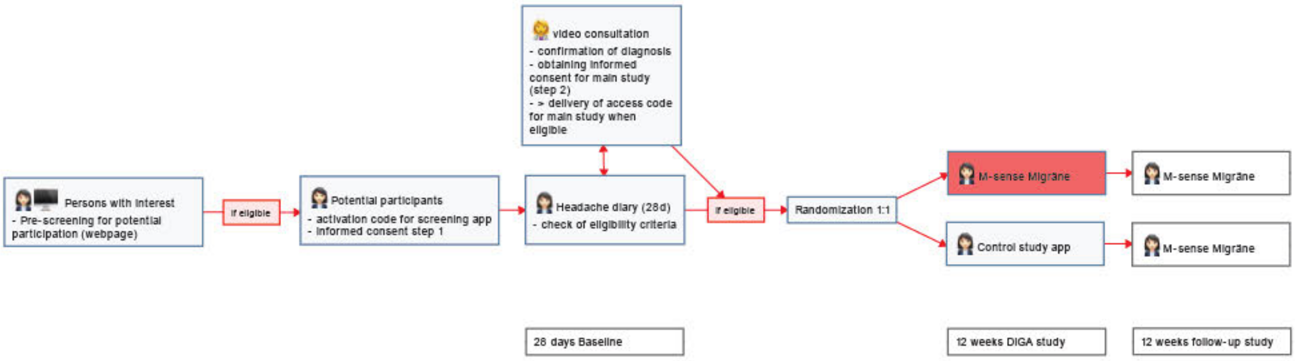


**Figure 1. Study flow and design of RCT**

## 4.2 Justification of study design and approach

The study takes into account the current guidelines of the International Headache Society for controlled trials on preventive pharmacological treatment of episodic migraine[18] and preventive treatment of chronic migraine[19] in adults. We decided to perform a randomized controlled trial to reduce the confounding of results as much as possible. Because of the type of intervention used within the study

blinding of participants is not possible. Nevertheless, the physician classifying the headache attacks and the statistician performing the analysis will blinded for the group allocation of the patients. To assure high relevance of the study results to patients, a patient reported outcome (PRO) was used as primary endpoint. We decided to document all outcomes within the app in both groups to reduce the bias that different types of documentation in intervention and control group might introduce to the data.

Although we assess most of the relevant diagnostic criteria within the app we decided to have the verification of the diagnoses by a physician. To stay within the digital framework of the study approach and taking into account possible restrictions because of the new corona virus SARS-CoV-2 this is planned as video consultation.

The DiGA study is planned for 12 weeks per patient (until primary endpoint), to gather further data and to allow the control group to receive the M-sense Migräne app after 12 weeks, a 12 week follow-up study will be added to the DiGA study.

# 5 PARTICIPANTS AND GROUP ALLOCATION

## 5.1 Recruitment

The study will be announced to possible participants and health professionals using different media channels. Additionally, participants can be recruited by self-referral via the German Apple App store (Apple Inc.) and Google Play Store. The recruitment of 346 patients within 6 months seems feasible, because in the last app study by the study group around 350 participants were recruited within 10 months only through the German Apple App store without applying other recruitment approaches.[11] Additionaly, this study will also recruit Android users. To support faster recruitment a fee of 80 Euro will be provided to study participants for a 12-week documentation within the app.

## 5.2 Inclusion and exclusion criteria

For inclusion in the study the patients have to fulfill the following **inclusion criteria**:

- Adults ≥ 18 years with migraine (ICD-10 G43) diagnosed by a physician
- Disease duration of at least 1 year (patient reported)
- At least 3 migraine days in the previous 4 weeks (baseline)
- Disease onset before age 50 y
- Migraine screening questions positively answered
- Smartphone ownership and smartphone literacy
- Sufficient knowledge of German

- Informed consent

Patients will be excluded from the study if they fulfill any of the following **exclusion criteria**:

- Planned pregnancy, pregnant and breastfeeding women (due to associations between hormone status and headache and migraine days)
- Medication overuse headache (assessed by physician question, app data during baseline > 15 days with medication)
- Headache app / M-sense or similar app use of at least a month during the last 12 months
- Planned start of a new treatment for migraine within the next 4 months
- Simultaneous participation in another interventional study
- Users who have filled in the diary <3 days/week during baseline

Inclusion and exclusion criteria will be assessed by a physician via video consultation, and within the app before final study entry after the 28 days baseline phase.

## 5.3 Verification of diagnosis

The diagnosis of will be verified by a physician via a video consultation and will also be checked by in-app questions before randomization.

## 5.4 Assignment to study groups

We will use a server-based randomization table implemented in the app and created by a statistician using the RANUNI random number generator of the SAS/STAT version 9.2 (SAS Inc).[20] Study participants fulfilling the eligibility criteria who have provided informed consent will be randomized in a 1:1 ratio by block randomization with a fixed block length to one of two groups (M-sense Migräne app or control group with control app). Randomization will be stratified for the type of migraine (episodic or chronic). To assure allocation concealment the randomization list will not be accessible by the user or the study staff involved in possible communication with the participants.

## 5.5 Withdrawal of study participants from treatment or assessment

Study participants have the right to abort the study at any time and without providing a reason. They can decide if they a) do not want to participate in the upcoming interventions and still want to complete questionnaires or b) do not want to participate in the upcoming interventions and do not want to complete

upcoming questionnaires or c) if they are withdrawing their informed consent and all previous information should be deleted. We will assess reasons for withdrawal.

In case of safety concerns, the patient will find information in the app on how to contact the study office where these aspects can be discussed. If general safety concerns arise, the study will be stopped and participants informed via the app and by the contact information they had provided.

# 6 OUTCOMES

## 6.1 Summary of assessments

Table 1 provides an overview about the assessments and the time points. The data is documented with the app.

**Table 1. Outcome and other assessments and time points, *documented by physician, #in app user data**

| Time (week) | Baseline 4 weeks | before randomization | Diary 12 weeks DIGA + 12 weeks after | 4 | 8 | 12 | 24-weeks for follow-up study |
|---|---|---|---|---|---|---|---|
| | | | | weeks | | | |
| Eligibility criteria | x | x* | | | | | |
| Migraine or chronic migraine diagnosis | x | x* | | | | | |
| Participant characteristics | x | x* | | | | | |
| Migraine days | x | | x | | | | |
| Headache days | x | | x | | | | |
| Moderate/severe headache days | x | | x | | | | |
| HIT-6 | | x | | | | x | x |
| HMSE-G-SF | | x | | | | x | x |
| HALT-30 | | x | | | | x | x |
| Migraine specific health literacy | | x | | | | x | |
| Medication use | x | | x | | | | |
| Use of app interventions | | | x# | | | | x# |
| Safety measures | | | | x | x | x | x |

## 6.2 Primary outcome

The primary outcome are the **migraine days per month** (defined as 28 days) after 12 weeks (week 9 to 12). Migraine days will be based on the information in the headache diary. Headache attacks will be classified according to ICHD-3[21] (criteria B to D) by a physician blinded for the patient's group allocation (see SAP 2.1 for further description). A migraine day is defined as a calendar day on which a single or multiple migraine attacks have been reported.

## 6.3 Secondary outcomes

The secondary outcomes include:

a) Migraine days per month (defined as 28 days) week 1 to 4 and week 5 to 8 calculated from the diary of the app.

b) Headache days and moderate/severe headache days per months (defined as 28 days) after 12 weeks (week 9 to 12), week 1 to 4, and week 5 to 8 will be calculated from the diary of the app.

c) Migraine specific quality of life measured with the **6-item Headache Impact Text (HIT-6)** over the last 4 weeks. The HIT-6 was designed to provide a global measure of adverse headache impact[22 23] and was developed to use in screening and monitoring patients with headaches in both clinical practice and clinical research.[22] The HIT-6 is a valid tool for both migraine and chronic migraine.[24]

d) Self-efficacy is measured with the 6-item short form of the German language adapted **headache management self-efficacy scale (HMSE-G-SF)**[25] over the last 4 weeks. The HSME-G-SF was designed to measure self-efficacy beliefs. The HSME-G-SF is a valid and reliable tool for migraine, chronic tension-type headache, or a combination of 2 or more headache disorders.

e) Headache attributed burden such as disability and headache attributed lost time is measured with the 5-item **Headache-Attributed Lost Time indices (HALT-30)**[26] over the last 4 weeks.

f) Migraine-specific health literacy will be evaluated. Because no suitable validated measure is available we will use a self-developed questionnaire with three items. The questionnaire is intended to assess the capability of migraineurs to find, understand, and judge information about the disease and treatment options.

g) **Drug consumptions** for acute treatment is measured from the medication diary within the app over the last 4 weeks on daily basis.

## 6.4 Further measurements

In addition to the above listed outcomes the following aspects will be measured:

a) Use of interventions

b) Safety: Serious Adverse Events, Suspected Adverse Reactions and Serious Suspected Adverse Reactions (for more details see chapter safety).

## 6.5 Mapping of outcomes to DVG categories

In the Digital Healthcare Act (DVG)[15] the concept of positive care effect was introduced. Positive healthcare effects (positive Versorgungseffekte) are defined as either a medical benefit (medizinischer Nutzen) or a patient-relevant improvement of structure and processes (patientenrelevante Struktur- und Verfahrensverbesserungen) in healthcare. The outcomes selected for this study were categorized into one of these two categories (see

Table **2**).

**Table 2. Mapping of outcomes according to DVG**

| Outcome | DVG Category | Notes |
|---|---|---|
| Migraine days (diary) | Medical Benefit - improvement of the state of health | Requested by the regulatory body (BfArM) and based on guidelines of the International Headache Society for controlled trials on preventive (pharmacological) treatment.[18 19] |
| Headache days (diary) | Medical Benefit - improvement of the state of health | Hint from the systematic secondary data analyses of M-sense Active, outcome of many migraine drug studies, e.g. also in AMNOG studies for monoclonal antibodies. |
| Migraine specific quality of life - HIT-6 | Medical Benefit – Health-related quality of life | Classic outcome of migraine drug & behavioural training studies, assessing migraine specific quality of life. |
| Self-efficacy HMSE-G-SF | Patient-relevant improvement of structure and processes - Patient Autonomy | Standard measure in the evaluation of behavioral intervention programs in migraine research. Measures whether patients' autonomous health behaviour is improved. |
| Disability and headache attributed lost time - HALT-30 | Medical Benefit - Health-related quality of life | Adapted and validated version of MIDAS (classic outcome in migraine research) with retrospective focus on 30 instead of 90 days, assessment of burden of disease. |
| Migraine-specific health literacy | Patient-relevant improvement of structure and processes - Health Literacy | Self-developed questionnaire |

# 7 INTERVENTION

## 7.1 Intervention group

M-sense Migräne app consists of the following evidence-based features and interventions. The evidence base has been mentioned in the introduction. Furthermore, it has been described in the systematic evaluation of M-sense Active user data where also the details of the app intervention are described. Here, this list of features and interventions will be provided:

a) Headache reporting and attack classification diary

b) Medication intake diary

c) Trigger factors diary

d) Analysis function and physician report

e) Personalized education on disease- and trigger management

f) Relaxation therapy

g) Training module

h) Acute pain therapy

i) Behavioral Change Techniques

The figure below gives some examples:

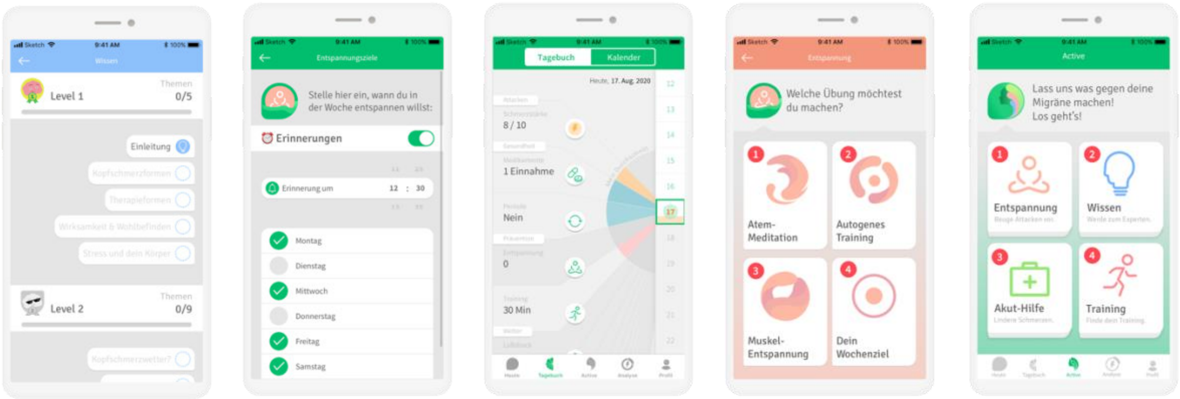


**Figure 2. Screenshots of the study app**

a) The comprehensive **headache diary** allows a real time tracking of headache occurrences, including headache characteristics, attendant symptoms and experienced pain intensity levels. These headache entries are automatically classified by the M-sense headache classification algorithm, a digitalized version of the ICHD-3-based headache classification.[21] The algorithm allocates single headache attacks to the phenotypes migraine, tension-type headache (TTH), and unclassified headaches.[27] The validity of M-sense's algorithm was assessed by comparing the classification of single headache attacks performed by the algorithm to the attack classification performed by a certified neurologist specialised in headache medicine. The substantial level of agreement between the neurologist and the algorithm was obtained (unweighted kappa=0.74). Most cases of disagreement were due to inadvertent mistakes of the neurologist as identified in the disagreement validity assessment.

b) The **diary for medication intake** allows users to track intake of their prophylactic and acute pain medication and to receive information on proper intake of medications and the potential hazards of medication overuse.

c) The **diary for trigger factors** list potential migraine trigger factors[28], e.g. sleep duration, period, alcohol consumption, stress (for details see Wöber & Wöber-Bingöl, 2011).[29] Through descriptive statistics potential individual triggers can be detected.

d) The **analysis function and physician report** analyses and visualizes the data entered into the app, including headache types, monthly headache occurrence, monthly average pain intensity and frequency of headaches, trigger statistics, headache distribution by time-of-day, day-of-week, and days-in-month and medication intake in weekly and monthly reports, as well as via the in-app calendar. This helps users to self-monitor the course of the disease and their medication intake. The report can be used to facilitate patient-physician communication.

e) For the **personalized education about disease- and trigger management** the M-sense Migräne app includes 20 individualized knowledge lessons about migraine (e.g. symptoms, trigger factors and causes) and its treatment options (acute and preventive).

f) For the **relaxation therapy** the app includes several relaxation exercises, such as Progressive Muscle Relaxation, Autogenic Training and Breathing Meditation, with different session lengths. To support behavioral self-management users can set reminders for relaxation therapy.

g) In the **training module** information on various suitable types of exercise and instructions on how to start practicing them adequately are provided. In addition, the users are supported to set up training plans. To support behavioral self-management users can set reminders for endurance sports.

h) For **acute pain therapy** M-sense Migräne incorporates non-pharmacological interventions, this includes animated physiotherapy exercises such as self-massage with a focus on migraine-specific pericrainial and neck muscular trigger points and stretching exercises, as well as guided visual imagery audio files.

i) **Behavioral Change Techniques** are used to support intervention engagement and behavioral self-management strategies. A monthly calendar gives an overview of successfully carried out preventive exercises, which prompts self-monitoring. To promote action planning, users can set up weekly plans with reminders for endurance sports and relaxation exercises. Education lessons as well as home-screen messages are personalized by means of diary entries, name, gender and age.

## 7.2 Control group

The control group will gain access to the control study app, an app configuration of the M-sense Migräne app. The control study app will serve as a data input tool and, thus, only include features for study data collection such as a limited headache and medication intake diary and questionnaires. The control study app will serve as a documentation "black box" that will not provide any feedback or interventions about their data entries or behavior. E.g. the user will not be shown the result of headache attack classification

or descriptive statistics about headache patterns. Headache entries can only be seen retrospectively for one week. The documentation within the app assures that both groups have the same way to document outcomes and avoids possible bias by digital placebo effects.[30]

## 7.3 Concomitant treatments

Concomitant treatments are allowed. Existing interventions and the start of new interventions will be documented within the app.

# 8 SAFETY

## 8.1 Risk-benefit assessment

The app M-sense Migräne is a CE marked class I medical device according to Annex IX of the Medical Device Directive (MDD 93/42/EEC) with the conformity assessment route MDD Annex VI. M-sense Migräne will be used within the intended purpose. The app is subject to a risk management process according to ISO 14971. It includes non-pharmacological evidence-based interventions for migraine[30] and is not expected to involve significant risks or burdens for the participants. We expect that the benefits will outweigh the risks. In a previous study within a project funded by the Innovation Fund (SMARTGEM)[31] the intervention was not associated with serious adverse events. M-sense Migräne fulfills the requirements of the Digital Health Applications Ordinance regarding Annex 1 (privacy and data security) as well as Annex 2 (robustness, consumer protection, patient safety, quality of medical content, support of healthcare providers, usability and accessability, and interoperability). However, misuse of data or a data breach could violate the patient’s privacy. These risks are reflected in the manufacturer’s privacy impact assessment and mitigation measurers are implemented. Each study participant will be informed about the applied interventions as well as about possible risks regarding privacy and data safety prior to study participation.

# 9 ASSESSMENT OF SAFETY

M-sense Migräne is a CE marked medical device with an established risk management which will also take place during the study period.

Safety outcomes defined for the study are the number of participants with serious adverse events, suspected adverse reactions and serious suspected adverse reactions.

All suspected adverse reactions and all serious adverse events will be listed and evaluated. An adverse event is any undesirable experience associated with the use of a therapy in a patient. The event is serious and should be reported when the patient outcome is: results in death or is life-threatening, requires inpatient hospitalization or prolongation of existing hospitalization, results in persistent or significant

disability/incapacity, or results in a congenital anomaly/birth defect (Good-Clinical-Practice-Verordnung, GCP-V)[32].

A suspected adverse reaction[33] in our study is defined as an effect that the app user directly associated (self reported) with the app use. For "serious" the same categories mentioned above are used.

In this study, patients will be asked about suspected adverse reactions and serious adverse events within the app (see Table 3).

In addition, we will collect serious suspected adverse reactions (patients will be informed during infomed consent and by the app to call the study office in case they assume to have a serious suspected adverse reactions).

A causality assessment of the serious adverse event with respect to the trial intervention (see Table 3 based on the terms given in the ICH E2A guideline[34]) will be performed. Any event assessed as possibly, probably or definitely related is classified as related to the trial intervention.

**Table 3. Tool for causality assessment of serious adverse events**

| Relationship | Description |
|---|---|
| Definitely | Temporal relationship<br>Improvement after dechallenge*<br>Recurrence after rechallenge<br>(or other proof of drug cause) |
| Probably | Temporal relationship<br>Improvement after dechallenge<br>No other cause evident |
| Possibly | Temporal relationship<br>Other cause possible |
| Unlikely | Any assessable reaction that does not fulfil the above conditions |
| Not related | Causal relationship can be ruled out |
| *Improvement after dechallenge only taken into consideration, if applicable to reaction | |

In addition, a severity assessment of the event (mild, moderate or severe) will be perfomed. Mild means the complication is tolerable, moderate means it interferes with daily activities and severe means it renders daily activities impossible.

Serious adverse event and suspected adverse reaction (patient reported) will be documented in an Serious adverse event log and evaluated by the study team every 3 months.

In case of serious suspected adverse reactions users call the study office and appropriate measures will be taken within 48 hours.

# 10 STUDY PROCEDURES

## 10.1 Pre-screening and pre-study screening log

The pre-screening of potential study participants will be carried out by a web page. In case of eligibility an app access code is provided. Every participant who shows interest in the study on the webpage is anonymously counted in a digital pre-study screening log. Furthermore, every participant who fulfills the prescreening criteria and receives a code to complete a 28 days baseline in the M-sense control app is anonymously counted in a digital pre-study screening log. The anonymous data will be used to describe the participant selection in a flow chart.

## 10.2 Baseline and informed consent

With the access code the control app can be used for a 28 days baseline assessment as recommended for migraine studies.[35] The participants will receive privacy information within the app and their electronic consent will be obtained.

The data will be used at the end of the baseline phase to assess the eligibility for the study. Close to the end of the baseline phase the migraine diagnosis will be confirmed in a video consultation by a physician, the main study eligibility will checked and the informed consent will be obtained. The informed consent includes general information about the study, inclusion and exclusion criteria, the course of the study, the study tasks, data gathering and data use, the risks, privacy, time commitment, and the withdrawal procedures. Eligible patients who provided informed consent will receive a study code from the physician. After they completed the baseline phase the control app checks the in-app eligibility criteria (migraine ≥ 3, filling in of the diary, no medication overuse). All other eligibility criteria will be checked by the physician. Participants who do not fulfill eligibility criteria will be offboarded by the app and their data will be deleted.

## 10.3 Randomization

Eligible participants will then be asked by the app to enter the study code received from the physician and will be randomized within the app. They participate in the DiGA study for 12 weeks according to their treatment allocation. After the end of the DiGA study the intervention group keeps the M-sense Migräne app and the control will also receive access to all the features of the M-sense Migräne app. Both groups will participate in a 12-weeks in a follow-up study.

## 10.4 Procedures in case of pregnancy

Although pregnant women will not be included into the study because hormonally change can influence the headache and migraine days, patients who become pregnant during the study period will not be excluded. This decision was made because we use and intention-to-treat population for the analysis and the interventions within the app are not known to have risks for pregnant women.

## 10.5 Discontinuation of the study

The study will be discontinued if less than 10% of the anticipated number of participants are recruited within the recruitment time.

# 11 STATISTICAL METHODS AND DETERMINATION OF SAMPLE SIZE

## 11.1 Sample size calculations

The following assumptions as basis for the sample size estimation are based on a pilot project: reduction of migraine days in the intervention group 1.69 migraine days, and in the control group 0.42 migraine days. This resulted in a group difference of 1.27 migraine days in week 9-12 between both groups with a pooled standard deviation of 4.20. Applying a 2-sided T-test for independent groups with a power of 80% and an alpha of 5%, a total of 173 participants will be needed per group (346 participants in total for both arms). Because the primary analyses will follow the intention-to-treat (ITT) principle, all randomized patients will be included and missing data will be imputed, additional patients to compensate for dropouts have not be taken into account. The sample size was calculated with the software PASS 2008 (Version 08.0.16, Release 27.01.2011; Hintze, J. (2008), NCSS, LLC, Kaysville, Utah). The rational for the assumptions has been described in the SAP (Version 2.1).

## 11.2 Analysis plan

A detailed statistical analysis plan (SAP) has been written (version 1.0 November 22, 2020) and accompanies this evaluation concept. For details please refer to the SAP.

## 11.3 Primary comparison and hypotheses

The primary analysis is a comparison of the group receiving the M-sense Migräne app with the group receiving the control app for the primary outcome (migraine days in weeks 9-12).

The following hypotheses will be tested:

$H_0$: the migraine days in week 9-12 are **not** different between the intervention and the control groups

In case $H_0$ can't be confirmed $H_A$ will be adopted

$H_A$: the migraine days in week 9-12 are different between the intervention and the control groups

## 11.4 Analysis populations

Based on the ITT principle, participants will be analysed in the which they were randomized, and we will include all patients in the analyses. This and other populations have been described in the SAP.

## 11.5 Participants demographics and baseline characteristics

All available baseline data will be analysed descriptively per group and in total. Results will be reported as means with standard deviations or as frequencies and percentages.

## 11.6 Primary outcome definition

The primary outcome are the **migraine days per month** (defined as 28 days) after 12 weeks (week 9 to 12). Migraine days will be based on the information in the headache diary. Headache attacks will be classified according to ICHD-3[21] (criteria B to D) by a physician blinded for the patient's group allocation (see SAP 2.1 for further description). A migraine day is defined as a calendar day on which a single or multiple migraine attacks have been reported. The number of migraine days per month is obtained by adding up the number of migraine days within four consecutive weeks (28 days).[36]

## 11.7 Analysis of the primary outcome

The analysis for the primary endpoint (migraine days week 9-12) will be conducted using an analysis of covariance (ANCOVA) with the treatment group (intervention/control) as fixed factor and baseline migraine days and type of migraine (episodic/ chronic as stratification variable during randomization) as covariates. From this model, estimated means and 95% confidence intervals will be obtained. Missing data will be imputed by using adequate multivariate imputation methods. In case of relevant differences in baseline variables between the treatment groups, the analysis of the primary outcome will be repeated with the additional adjustment for these variables.

## 11.8 Secondary outcomes

Secondary outcomes will be analysed similarly to the primary analysis, i.e. ANCOVA, or logistic or Poisson regression (depending on the scale and distribution of the data), adjusted for sex and the respective baseline value (when available). Detailed description of all analyses will be given in the SAP.

All analyses on secondary outcomes will be considered explorative.

## 11.9 Further analyses

As supportive analysis models for repeated measures will be fitted to compare the groups with respect to changes in the primary outcome over time. Further details will be described in the SAP.

## 11.10 Analyses of safety outcomes

All safety variables will be tabulated (in total and per treatment group). Reported are number of participants with suspected adverse reactions, serious suspected adverse reactions,  and adverse events, number of participants who withdraw from the study due to suspected adverse reactions. Categorical variables are reported using frequency and percentage, continuous variables are reported by giving descriptive measures (mean, standard deviation/standard error, quartiles, and range). The evaluation of safety endpoints is performed with the safety population.

## 11.11 Interim analyses

No interim analyses are planned.

# 12 QUALITY CONTROL AND QUALITY ASSURANCE

## 12.1 Data Management

The trial will be conducted according to the Standard Operating Procedures (SOPs) and the quality management protocols of the Institute for Social Medicine, Epidemiology, and Health Economics and of Newsenselab. Newsenselab is responsible for the central storage of the collected data and will transfer the data adequately encrypted and via a safe transfer method as a csv-file to the study investigators. The data are coded with the EMMA-ID (pseudonymization) rather than with clear names, so that it is not possible to assign them directly to the study participants. The access to the linked person identifying data (such as name and contact information) is restricted (physician who obtains informed consent and defined personal of the study office at the Charité). Study investigators will work with aggregated pseudonymized data and will only publish data in anonymized form. Data received from Newsenselab will be checked for plausibility, correctness, and completeness. After a blind review data will transposed to a SPSS file or similar format for statistical analysis.

## 12.2 Data monitoring

M-sense Migräne's data validity is established as a CE marked medical device and reviewed regulary as part of its quality management system. An audit trail log covering the data trail from each participant's

data entry to the export will be reviewed by the study investigators. Further procedures to test data validity will be established by the investigators.

# 13 ETHICS AND REGULATIONS

This study will follow the standards of the Helsinki Declaration (1996 version, Somerset West) and will be based on ICH-GCP guidelines. The study is approved by the responsible ethics committee (Charité – Universitätsmedizin Berlin; EA1/343/20). The study is also subject to review by the data protection officer of the Charité – Universitätsmedizin Berlin. The study participants have the right to obtain information about the data collected about them at any time. Participation in the study will be voluntary and will only take place after written consent has been given following detailed explanation of the study contents and objectives. Participation in the study can be terminated by the patient at any time without any disadvantages for the patient.

The study will be conducted according to § 23 b MPG (Medizinproduktegesetz) – exemption of clinical investigation. Although randomized the study follows a routine care setting usage of the app. There are no invasive study procedures and most of the documentation for the study has been already implemented as a routine care measure of the app (e.g. diary). The physician visit for the diagnosis reflects a typical diagnostic case taking of migraine patients. The M-sense Migräne app will be used only within the intended purpose.

EU General Data Protection Regulation (GDPR) as well as the Berlin and the Federal Data Protection Acts are applied. Both Newsenselab GmbH and the Institute for Social Medicine, Epidemiology and Health Economics have established quality management systems. A privacy policy for the study and the app will be available. We will collect only data which is necessary for the study. The access to the linked person identifying data (such as name and contact information) is restricted (physician who obtains informed consent and defined personal of the study office at the Charité). Study data is stored encrypted on Newsenselab's servers during the study. Any data transfer between the app and the servers is state-of-the-art encrypted. A detailed data protection concept for the study will be developed based on the SMARTGEM[31] study funded by the Innovation Fund.

## 13.1 Insurance

No special insurance is purchased for the patients for this study. The Charité employees involved in the study are insured by the public liability insurance of Charité – Universitätsmedizin Berlin against liability claims that could result from their culpable behavior.

## 13.2 Study registration

This trial will be registered after ethics and BfArM approval in the German Register for Clinical Trials (DRKS; https://www.drks.de/drks_web/).

# 14 PUBLICATION

Results of the study will be made publicly available within 12 months after end of the study, if necessary as pre-print. We will submit the results of the study to peer-reviewed international journals. The chances of a manuscript being accepted by a medium to high ranked journal are considerable, presuming that the results of the study are conclusive.

# 15 STUDY SCHEDULE

The study will follow the following schedule:

- Baseline First-Participant-In: January 1, 2021
- Study First-Participant-In: January 29, 2021
- Baseline Last-Participant-Out: June 30, 2021
- Study Last-Participant-Out: Sep 30, 2021
- Data Analysis and report to BfArM: Dec 15, 2021

# 16 REFERENCE LIST

1. Stewart WF, Shechter A, Rasmussen BK. Migraine prevalence. A review of population-based studies. *Neurology* 1994;44(6 Suppl 4):S17-23. [published Online First: 1994/06/01]

2. Silberstein SD. Migraine. *Lancet* 2004;363(9406):381-91. doi: 10.1016/S0140-6736(04)15440-8 [published Online First: 2004/04/09]

3. Silberstein SD. Preventive Migraine Treatment. *Continuum (Minneap Minn)* 2015;21(4 Headache):973-89. doi: 10.1212/CON.0000000000000199 [published Online First: 2015/08/08]

4. Estemalik E, Tepper S. Preventive treatment in migraine and the new US guidelines. *Neuropsychiatr Dis Treat* 2013;9:709-20. doi: 10.2147/NDT.S33769 [published Online First: 2013/05/30]

5. Kumar A, Kadian R. Migraine Prophylaxis. StatPearls. Treasure Island (FL)2020.

6. Whyte CA, Tepper SJ. Adverse effects of medications commonly used in the treatment of migraine. *Expert review of neurotherapeutics* 2009;9(9):1379-91. doi: 10.1586/ern.09.47 [published Online First: 2009/09/23]

7. Martin PR. Behavioral management of migraine headache triggers: learning to cope with triggers. *Curr Pain Headache Rep* 2010;14(3):221-7. doi: 10.1007/s11916-010-0112-z [published Online First: 2010/04/29]

8. Puledda F, Shields K. Non-Pharmacological Approaches for Migraine. *Neurotherapeutics* 2018;15(2):336-45. doi: 10.1007/s13311-018-0623-6 [published Online First: 2018/04/05]

9. Michie S, Richardson M, Johnston M, et al. The behavior change technique taxonomy (v1) of 93 hierarchically clustered techniques: building an international consensus for the reporting of behavior change interventions. *Ann Behav Med* 2013;46(1):81-95. doi: 10.1007/s12160-013-9486-6 [published Online First: 2013/03/21]

10. Michie S, Abraham C, Eccles MP, et al. Strengthening evaluation and implementation by specifying components of behaviour change interventions: a study protocol. *Implement Sci* 2011;6:10. doi: 10.1186/1748-5908-6-10 [published Online First: 2011/02/09]

11. Wang J, Rogge AA, Armour M, et al. International ResearchKit App for Women with Menstrual Pain: Development, Access, and Engagement. *JMIR Mhealth Uhealth* 2020;8(2):e14661. doi: 10.2196/14661 [published Online First: 11.2.2020]

12. Michie S, Johnston M. Behavior Change Techniques. In: Gellman MD, Turner JR, eds. Encyclopedia of Behavioral Medicine. New York, NY: Springer New York 2013:182-87.

13. DeSmet A, De Bourdeaudhuij I, Chastin S, et al. Adults' Preferences for Behavior Change Techniques and Engagement Features in a Mobile App to Promote 24-Hour Movement Behaviors:

Cross-Sectional Survey Study. *JMIR Mhealth Uhealth* 2019;7(12):e15707. doi: 10.2196/15707 [published Online First: 2019/12/21]

14. Mosadeghi-Nik M, Askari MS, Fatehi F. Mobile health (mHealth) for headache disorders: A review of the evidence base. *J Telemed Telecare* 2016;22(8):472-77. doi: 10.1177/1357633X16673275 [published Online First: 2016/11/02]

15. BfArM. Digitale-Versorgung-Gesetz / Medical Apps 2020 [Available from: https://www.bfarm.de/DE/Medizinprodukte/DVG/_node.html accessed 11.09.2020.

16. Bundesanzeiger. Bundesgesetzblatt 2020 [Available from: https://www.bgbl.de/xaver/bgbl/start.xav?startbk=Bundesanzeiger_BGBl&jumpTo=bgbl119s2562.pdf#__bgbl__%2F%2F*%5B%40attr_id%3D%27bgbl119s2562.pdf%27%5D__1581721908992 accessed 17.02.2020.

17. M-sense. Übernimm die Kontrolle bei Migräne & Kopfschmerzen 2020 [Available from: https://www.m-sense.de/ accessed 17.02.2020.

18. Diener HC, Tassorelli C, Dodick DW, et al. Guidelines of the International Headache Society for controlled trials of preventive treatment of migraine attacks in episodic migraine in adults. *Cephalalgia* 2020;40(10):1026-44. doi: 10.1177/0333102420941839 [published Online First: 2020/07/30]

19. Tassorelli C, Diener HC, Dodick DW, et al. Guidelines of the International Headache Society for controlled trials of preventive treatment of chronic migraine in adults. *Cephalalgia* 2018;38(5):815-32. doi: 10.1177/0333102418758283 [published Online First: 2018/03/06]

20. SAS/STAT [program]. version 9.2 version. Cary, NC, USA., 2008.

21. Headache Classification Committee of the International Headache Society (IHS) The International Classification of Headache Disorders, 3rd edition. *Cephalalgia* 2018;38(1):1-211. doi: 10.1177/0333102417738202 [published Online First: 2018/01/26]

22. Kosinski M, Bayliss MS, Bjorner JB, et al. A six-item short-form survey for measuring headache impact: the HIT-6. *Qual Life Res* 2003;12(8):963-74. doi: 10.1023/a:1026119331193 [published Online First: 2003/12/04]

23. Ware JE, Jr., Bjorner JB, Kosinski M. Practical implications of item response theory and computerized adaptive testing: a brief summary of ongoing studies of widely used headache impact scales. *Med Care* 2000;38(9 Suppl):II73-82. [published Online First: 2000/09/12]

24. Yang M, Rendas-Baum R, Varon SF, et al. Validation of the Headache Impact Test (HIT-6) across episodic and chronic migraine. *Cephalalgia* 2011;31(3):357-67. doi: 10.1177/0333102410379890 [published Online First: 2010/09/08]

25. Graef JE, Rief W, French DJ, et al. German Language Adaptation of the Headache Management Self-Efficacy Scale (HMSE-G) and Development of a New Short Form (HMSE-G-SF). *Headache* 2015;55(7):958-72. doi: 10.1111/head.12564 [published Online First: 2015/04/24]

26. Steiner TJ, Lipton RB, Al Jumah M, et al. The Headache-Attributed Lost Time (HALT) Indices: measures of burden for clinical management and population-based research. *The Journal of Headache and Pain* 2018;19(1):12. doi: 10.1186/s10194-018-0837-3

27. Roesch A, Dahlem MA, Neeb L, et al. Validation of an algorithm for automated classification of migraine and tension-type headache attacks in an electronic headache diary. *J Headache Pain* 2020;21(1):75. doi: 10.1186/s10194-020-01139-w [published Online First: 2020/06/14]

28. Pavlovic JM, Buse DC, Sollars CM, et al. Trigger factors and premonitory features of migraine attacks: summary of studies. *Headache* 2014;54(10):1670-9. doi: 10.1111/head.12468 [published Online First: 2014/11/18]

29. Wöber C, Wöber-Bingöl Ç. Chapter 12 - Triggers of migraine and tension-type headache. In: Aminoff MJ, Boller F, Swaab DF, eds. Handbook of Clinical Neurology: Elsevier 2010:161-72.

30. Torous J, Firth J. The digital placebo effect: mobile mental health meets clinical psychiatry. *Lancet Psychiatry* 2016;3(2):100-2. doi: 10.1016/S2215-0366(15)00565-9 [published Online First: 2016/02/07]

31. Netzwerk SMARTGEM. Smartphone gestützte Migränetherapie als neue Versorgungsform 2020 [Available from: https://smartgem-projekt.de accessed 08-20-2020 2020.

32. German Federal Ministry of Justice. Verordnung über die Anwendung der Guten Klinischen Praxis bei der Durchführung von klinischen Prüfungen mit Arzneimitteln zur Anwendung am Menschen (GCP-Verordnung - GCP-V) 2004 [Available from: https://www.gesetze-im-internet.de/gcp-v/BJNR208100004.html accessed 01/02 2019.

33. Food And Drug Administration. Food and Drugs. Investigational New Drug Application. Safety Reporting, 2016.

34. ICH. ICH E2A Clinical safety data management: definitions and standards for expedited reporting. London: ICH, 1995.

35. Silberstein S, Tfelt-Hansen P, Dodick DW, et al. Guidelines for controlled trials of prophylactic treatment of chronic migraine in adults. *Cephalalgia* 2008;28(5):484-95. doi: 10.1111/j.1468-2982.2008.01555.x [published Online First: 2008/02/26]

36. Newsenselab GmbH. Systematic Data Analysis of M-sense Active User Data. Justification of the Improvement of Healthcare (Application for Provisional Listing). Berlin, 2020:56.

DRKS__________

# Statistical Analysis Plan (SAP)

# for the study:

# Effectiveness of the M-sense Migräne app – a randomized controlled study - EMMA Study

# DRKS_______

**Version: 2.1**

Datum: 09.12.2020

# SAP Revision History

| Previous Version | Updated Version | Sections changed | Description and reason for change | Date of change |
|---|---|---|---|---|
| | 0.0 | | Creation of document | 10.11.2020 |
| 0.0 | 1.0 | | First final version | 22.11.2020 |
| 1.0 | 2.0 | Sections 1, 2, 3, 5, 6.1, 6.2, 7, 8, 10.4.1-10.4.3, 10.5.1, 10.5.3, Table 2 | Change of primary outcome as requested by the regulatory authority (BfArM), specification of handling of missing data, adaption of sample size calculation | 05.12.2020 |
| 2.0 | 2.1 | Sections 3, 6.1 | Change of attack classification | 09.12.2020 |

Authors: Jürgen Barth[1] and Daniel Pach[1,2], Katja Icke[2], Claudia M. Witt[1;2]

[1]Institute for Complementary and Integrative Medicine, University Hospital Zurich and University of Zurich

[2]Institute for Social Medicine, Epidemiology and Health Economics, Charité – Universitätsmedizin Berlin

# SIGNATURES

**Sponsor**

[redacted]

| | |
|---|---|
| 09.12.2020 | |
| Date | Markus Dahlem |

**Principal investigator**

[redacted]

| | |
|---|---|
| 09.12.2020 | |
| Date | Claudia M. Witt, MD, [redacted] |

**Co-Principal investigator**

[redacted]

| | |
|---|---|
| 09.12.2020 | |
| Date | Daniel Pach, MD |

**Statistician**

[redacted]

| | |
|---|---|
| 09.12.2020 | |
| Date | PD Dr. phil. J[redacted]rgen B[redacted]arth [redacted] |

**Data manager**

[redacted]

| | |
|---|---|
| 09.12.2020 | |
| Location, Date | Katja Icke [redacted] |

# TABLE OF CONTENTS


**1 Introduction ........ 5**
**2 Aim of the trial ........ 5**
**3 Study design ........ 5**
**4 Intervention and control ........ 6**
**5 Changes to the study protocol ........ 6**
**6 Outcomes ........ 6**
**6.1 Primary Outcome ........ 7**
**6.2 Secondary Outcomes ........ 9**
**6.3 Further measurements ........ 10**
**6.4 Safety measurements ........ 10**
**7 Sample size calculation ........ 11**
**8 Primary comparison and hypothesis ........ 12**
**9 Statistical principles ........ 12**
**9.1 Confidence intervals and p-values ........ 12**
**9.2 Analysis populations ........ 13**
9.2.1 Intention-to-treat population (ITT) ........ 13
9.2.2 Full analysis set (FAS) ........ 13
9.2.3 Safety population ........ 13
**10 Statistical analyses ........ 13**
**10.1 Timing of analyses ........ 13**
**10.2 Statistical interim analyses ........ 13**
**10.3 Descriptive analyses ........ 13**
**10.4 Handling dropouts and missing data ........ 14**
10.4.1 Dropouts intervention group ........ 14
10.4.2 Dropouts in the control group ........ 14
10.4.3 Missing Data ........ 15
**10.5 Analysis for the effectiveness of the app ........ 15**
10.5.1 Primary analysis of the primary outcome ........ 15
10.5.2 Sensitivity analyses of the primary outcome ........ 16
10.5.3 Moderators and Subgroup Analyses ........ 16
10.5.4 Secondary outcomes ........ 17
**10.6 Analysis of adherence to the study intervention ........ 17**
**10.7 Analysis of safety ........ 17**
**11 Software ........ 17**
**12 References ........ 20**

# 1 INTRODUCTION

This is the Statistical Analysis Plan (SAP) for the digital health application (DiGA) study: 'Effectiveness of the M-sense Migräne app – a randomized controlled study - EMMA Study', that substitutes the evaluation concept (version 2.1). The study has been approved by the ethics committee of the Charité – Universitätsmedizin Berlin (EA1/343/20). This SAP describes only the analyses for the 12-weeks DiGA study period. An additional SAP for the 24-weeks follow-up study that is outside DiGA study will be developed in the first half of 2021.

# 2 AIM OF THE TRIAL

The aim is to evaluate the effectiveness of the M-sense Migräne app in reducing migraine days in patients with episodic migraine and chronic migraine compared to patients in a waiting list control group who have no access to the app intervention features.

# 3 STUDY DESIGN

This is a two-armed, open-label, parallel-group, randomized study. Patients will be randomly allocated to one of two groups (intervention or control as a waiting list) in a 1:1 ratio (stratified for episodic and chronic migraine) to gain access either to the intervention (M-sense Migräne app) or an app version with only data input features (control app). For motivational reasons the control group participants will automatically gain access to the M-sense Migräne app after 12 weeks. These additional data and the 24-weeks follow-up in the intervention group that uses M-sense Migräne from the beginning will not be part of the DiGA study.

Methodological considerations: When planning the trial and the data analyses we were aware that the patients cannot be blinded to the group they are allocated to, which might introduce bias. However, because of the type of intervention blinding is not possible and open-label trials are common in behavioral interventions. Nevertheless, the physician classifying the headache attacks and the statistician performing the analysis will blinded for the group allocation of the patients.

Because the number of migraine days is higher in patients with chronic migraine than in patients with episodic migraine, we use the specification of diagnosis as stratification variable during the randomization.

To reduce missing data for the primary outcome, patients are reminded within the app to complete the daily headache question and document their headaches thoroughly. Furthermore, we motivate

those patients who are non-adherent and stop the intervention to at least document their headaches within the app[1]. Based on this we can distinguish in the group using M-sense Migräne between study dropouts and intervention dropouts (see 10.4.).

# 4 INTERVENTION AND CONTROL

The intervention group receives the M-sense Migräne app in the DiGA study for 12 weeks. The app includes the following features: a) Headache reporting and attack classification diary; b) Medication intake diary; c) Trigger factors diary; d) Analysis function and physician report; e) Personalized education on disease- and trigger management; f) Relaxation therapy; g) Training module; h) Acute pain therapy, and i) Behavioral Change Techniques.

The control group will use within the 12 weeks an app ("control app") that only includes data input features (headache reporting and diary and medication intake diary) and questionnaires. The headache diary will not show any attack classification, analysis or aggregated reports on the documented data to the patient. The headache diary is limited to the last seven days, which means that patients cannot enter or edit headache attacks or medication intakes that date back seven days or longer. The control app does not contain any of the other abovementioned app interventions.

# 5 CHANGES TO THE STUDY PROTOCOL

This SAP substitutes the evaluation concept (version 2.1). Changes since version 1.0 of the evaluation concept are 1) classification of headache attacks by a physician instead of an algorithm, 2) the change of the primary endpoint headache days after 12 weeks to migraine days after 12 weeks as requested by the regulatory authority (BfArM), 3) the specification of the secondary outcome "severe headache" to moderate/severe headache days, because this is the commonly used one, and 4) the further measurement "concomitant interventions" to "concomitant activities / activities", because the questions include both activities recommended by the app and activities that are not included but might be used concomitantly.

# 6 OUTCOMES

Data for the DiGA study will be collected at different time points (baseline (for 4 weeks before randomization), 4 weeks, 8 weeks, and 12 weeks), for details see Table 1. Scale properties, range of possible values, and interpretation of values for each outcome are given in Table 2.

**Table 1.** Data collection

| Time (week) | Baseline 4 weeks | Before randomization | Diary 12 weeks DIGA study | 4 | 8 | 12 | + 12 weeks for follow-up study# |
|---|---|---|---|---|---|---|---|
| | | | | weeks | | | |
| **Eligibility criteria** | x | x | | | | | |
| **Episodic migraine or chronic migraine diagnosis** | x | x | | | | | |
| **Participant characteristics** | x | x | | | | | |
| **Migraine days** | x | | x | | | | |
| **Headache days** | x | | x | | | | |
| **Moderate/severe headache days** | x | | x | | | | |
| **HIT-6** | | x | | | | x | x |
| **HMSE-G-SF** | | x | | | | x | x |
| **HALT-30** | | x | | | | x | x |
| **Migraine specific health literacy** | | x | | | | x | |
| **Medication use** | x | | x | | | | |
| **Use of app interventions** | | | x | | | | x |
| **Safety measures** | | | | x | x | x | x |

#not part of this SAP

## 6.1 PRIMARY OUTCOME

The primary outcome are the **migraine days per month** (defined as 28 days) after 12 weeks (week 9 to 12). Migraine days will be based on the information in the headache diary. Headache attacks will be classified according to ICHD-3[2] (criteria B to D) by a physician blinded for the patient's group allocation. A migraine day is defined as a calendar day on which a single or multiple migraine attacks have been reported.

The information necessary for the classification as migraine attack ICHD-3 criteria B to D (see below) will be used based on data of the diary.

The criteria B to D are[2]:

B. Headache attacks lasting 4-72 hr (untreated or unsuccessfully treated)
C. Headache has at least two of the following four characteristics:
    1. unilateral location
    2. pulsating quality
    3. moderate or severe pain intensity
    4. aggravation by or causing avoidance of routine physical activity (eg, walking or climbing stairs)
D. During headache at least one of the following:
    1. nausea and/or vomiting
    2. photophobia and phonophobia

The physician will manually classify the headache attacks based on the raw data of the diary according to ICHD-3[2] (criteria B to D). Any information which has to be manually added to the analysis dataset will be based on double entry and subsequent comparison to control for data entry errors. In a next step we will compare the results from the manual classification with the M-sense algorithm[3]. In case of discrepancies the physician will re-evaluate his/her classification and make a final decision.

The number of migraine days per month is obtained by adding up the number of migraine days within four consecutive weeks (28 days).[4] When the respective information needed for the classification has been sufficiently documented by the patient the headache days will be classified into three categories: 1. migraine days, 2. days with tension type headache, or 3. other headaches (do not meet criteria for migraine days or days with tension type headache). If the respective information provided by the patient is not sufficient for classification, the classification value for this day would be seen as a missing value.

To support a complete documentation technically, a daily headache question with a dichotomous value (headache yes/ no) has been implemented in both app versions (M-sense Migräne app and control app). This question will be shown every day when the app is opened for the first time. The app also motivates patients to open the app daily and document their headache attacks. The headache diary records the time period of attacks, as well as the pain characteristics and attendant symptoms for every documented headache attack. The choice of the primary outcome was requested by the regulatory body (BfArM) based on the Guidelines of the International Headache

Society for controlled trials on preventive (pharmacological) treatment of episodic migraine[5] and preventive treatment of chronic migraine[6] in adults.

## 6.2 SECONDARY OUTCOMES

Secondary outcomes based on the diary within the app:

- Migraine days per month (months defined as 28 days) week 1 to 4 and week 5 to 8.
- Headache days per month (months defined as 28 days) week 1 to 4, week 5 to 8, and week 9 to 12. The headache days are defined as the number of days on which headaches have been documented by the patient in the diary. The number of headache days per month is then obtained by adding up the number of days on which headaches have been reported within four consecutive weeks (28 days).[4]
- Moderate/severe headache days (defined as a headache that is longer than 4 hours and is accompanied with a pain severity of at least 4, as reported on the in-app 11-point numeric rating scale (NRS) for pain, 0=”kein Schmerz” to 10=”stärkster Schmerz”) per months (defined as 28 days) after 12 weeks (week 9 to 12). Furthermore, we will analyze week 1 to 4 and week 5 to 8.
- Treatment responder defined as at least a 30% reduction in migraine days per month (defined as 28 days) after 12 weeks (week 9 to 12) compared to baseline. Study dropouts (see 10.4 for definition) will be handled as non-responders in the analysis.
- Drug consumption for acute pain treatment includes: a) days with medication for acute pain, and b) days with triptane use. This is documented in the daily medication diary (week 1-12).

Secondary outcomes based on questionnaires within the app:

- Migraine specific quality of life measured with the 6-item Headache Impact Text (HIT-6)[7,8] over the last 4 weeks after 12 weeks. For the analyses we will aggregate the single items in an index using the The PRO CoRE™ Software and follow the “HIT-6: A User's Guide eManual” of the license holder Optum.
- Self-efficacy measured with the 6-item short form of the German language adapted headache management self-efficacy scale (HMSE-G-SF)[9] over the last 4 weeks measured after 12 weeks.

- Headache attributed burden such as disability and headache attributed lost time is measured with the 5-item Headache-Attributed Lost Time indices (HALT-30[10], official German translated Version[11]) over the last 30 days after 12 weeks. The HALT-30 was developed as part of "The Global Campaign against Headache" with support from the World Health Organization. The HALT items are fully based on the five MIDAS items with slightly changed wording for more clarity and a shorter retrospective reporting period (30 instead of 90 days). Because of this, the HALT-30 can be viewed as an improvement of the MIDAS and the indices show no changes to the structure and scoring of the well-validated MIDAS[12].
- Migraine-specific health literacy after 12 weeks (3 items, own development) with the items prophylaxis literacy, disease literacy, trigger literacy on a 4 point Likert-type scale from 1=”sehr gut” to 4= “sehr schlecht”.

## 6.3 FURTHER MEASUREMENTS

- Adherence to the study intervention is only observed in the M-sense Migräne app group and on weekly basis. Being adherent is defined for a patient as using an app feature which is not implemented in the control app, at least one time per week.
- Tracked usage of app features during the 12 weeks is only measured in the intervention group with M-sense Migräne including (a) Headache reporting and attack classification diary; b) Medication intake diary; c) Trigger factors diary; d) Analysis function and physician report; e) Personalized education on disease- and trigger management; f) Relaxation therapy; g) Training module; h) Acute pain therapy. This data will be displayed descriptively.
- Concomitant activities/ activities are documented in both groups (activities recorded in an app diary: mindfulness practice, breathing meditation, autogenic training, muscle relaxation, qigong/tai chi, yoga, nordic walking, cardio-training, fitness course, running, cycling, and swimming) used against headache during the first 12 weeks (activity, and time spent for respective activity). Some of these activities are recommended in the app, others not. We will calculate the number of patients with respective activities.

## 6.4 SAFETY MEASUREMENTS

In both groups:

- Number of patients with a serious adverse event during the first 4 weeks, second 4 weeks, and third 4 weeks, and in total.
- Counts of serious adverse events during the first 4 weeks, second 4 weeks, and third 4 weeks, and in total.

In the M-sense Migräne app group only:

- Number of patients with a suspected adverse reaction during the first 4 weeks, second 4 weeks, and third 4 weeks, and in total.
- Counts of suspected adverse reactions during the first 4 weeks, second 4 weeks, and third 4 weeks, and in total.
- Duration of suspected adverse reaction.

# 7 SAMPLE SIZE CALCULATION

We aim to recruit 346 study participants (173 per group). The sample size estimation is based on the comparison between the group receiving the M-sense Migräne app with the group receiving the control app version for the primary outcome (migraine days in weeks 9-12), which will be treated as a continuous variable.

For the assumptions of our sample size calculation we used diary data of a project that started in July 2020, (all 19 patients included until November 11, 2020, who used the app at least over a period of 56 days after inclusion). The "Region of the Future of Digital Health" (Zukunftsregion Digitale Gesundheit) is an initiative to position Berlin as a core region for digital health in Germany by creating a digital health ecosystem forested by the Digital Healthcare Act. With the app M-sense Migräne we have been selected to participate in the "ZDG" by public tender. We think that this data can serve as suitable pilot data for the EMMA study, because M-sense Migräne is used and physicians confirm the migraine diagnosis.

Since July 2020, physicians in Berlin who are involved in the project could prescribe M-sense Migräne to their patients after migraine diagnoses and patients complete the diary and can use the interventions. During the first 4 weeks the patients had a mean of 5.63 (sd 4.20) migraine days and in the second 4 weeks (3.94 (sd 4.18), resulting in a difference of 1.69 days.

However, because this project uses a pre-post design without a 4-week baseline before intervention we had to use the first 4 weeks of the intervention as baseline, and because the project just started

in summer only 4 further weeks were available. Both aspects will result in an underestimation of the effect but support a conservative approach of the assumptions.

In addition, we assume that using the app with no app intervention in the control group can also have an impact on the primary outcome but that this is smaller than in the intervention group. We assume a reduction of 25% of the migraine days of the intervention group in the control group (=0.42 days). Since the placebo effects described in the literature[13] for migraine prophylaxis were mainly around 20% of the effect, this seems to be reasonable conservative.

Based on this explanation we used the following assumptions as basis for the sample size estimation: reduction of migraine days in the intervention group 1.69 migraine days, and in the control group 0.42 migraine days. This resulted in a group difference of 1.27 migraine days in week 9-12 between both groups with a pooled standard deviation of 4.20. Applying a 2-sided T-test for independent groups with a power of 80% and an alpha of 5%, a total of 173 participants will be needed per group (346 participants in total for both arms). Because the primary analyses will follow the intention-to-treat (ITT) principle, all randomized patients will be included and missing data will be imputed, additional patients to compensate for dropouts have not be taken into account. The sample size was calculated with the software PASS 2008 (Version 08.0.16, Release 27.01.2011; Hintze, J. (2008), NCSS, LLC, Kaysville, Utah).

# 8 PRIMARY COMPARISON AND HYPOTHESIS

The primary analysis is a comparison of the group receiving the M-sense Migräne app with the group receiving the control app for the primary outcome (migraine days in weeks 9-12).

The following hypotheses will be tested:

$H_0$: the migraine days in week 9-12 are **not** different between the intervention and the control group

In case $H_0$ can't be confirmed $H_A$ will be adopted

$H_A$: the migraine days in week 9-12 are different between the intervention and the control group

# 9 STATISTICAL PRINCIPLES

## 9.1 CONFIDENCE INTERVALS AND P-VALUES

All confidence intervals are 95% and two-sided. All tests are two-sided and performed at a significance level of 0.05. The test statistics for the primary analyses is judged as confirmatory. All other tests are judged as explorative.

## 9.2 ANALYSIS POPULATIONS

### 9.2.1 Intention-to-treat population (ITT)

The intention-to-treat population (ITT) will consist of all randomized patients. Patients will be analyzed in the treatment group in which they were randomized. Handling of dropouts and missing values are described in chapter 10.4.

### 9.2.2 Full analysis set (FAS)

The full analysis set (FAS) will consist of all randomized patients with baseline and follow-up values available for the respective outcome analysis. Patients will be analyzed by the treatment group in which they were randomized.

### 9.2.3 Safety population

The safety population will include all patients according to the group of the type of app they used (M-sense Migräne or control app).

# 10 STATISTICAL ANALYSES

## 10.1 TIMING OF ANALYSES

The duration of the DiGA study is planned to be 16 weeks per patient (4 weeks baseline plus 12 weeks of intervention). About 14 weeks after the last patient has been randomized to the study groups, the clean file will be prepared to start the analysis of this DiGA study.

## 10.2 STATISTICAL INTERIM ANALYSES

No interim analyses have been planned.

## 10.3 DESCRIPTIVE ANALYSES

Enrolled, allocated, and analyzed patients will be reported following the standards of the CONSORT 2010 flow diagram[14,15].

All available baseline data will be analyzed descriptively per treatment group and in total. Interval and ratio variables will be reported as means, medians and standard deviations (SD), nominal and ordinal variables as frequencies and percentages.

Furthermore, we will analyze in the group with the M-sense Migräne app the tracked usage of app features during the 12 weeks.

## 10.4 HANDLING DROPOUTS AND MISSING DATA

### 10.4.1 Dropouts intervention group

Dropouts in the intervention group (randomized to the M-sense Migräne app) are defined as patients who are **not** answering the daily headache question at least 3 days per week for at least 4 weeks consecutively OR have been non-adherent to the intervention for at least 4 weeks consecutively (please see definition for adherence 6.3.). We distinguish between study dropouts and intervention dropouts and will employ different methods to handle missing data for the different types of dropouts in the group with the M-sense Migräne app.

- Intervention dropout are non-adherent for at least 4 consecutive weeks, BUT answer the daily headache question at least 3 days per week during the weeks of non-adherence. For this group missing data will only be imputed for the weeks where missing data was detected according to the missing data definition (see 10.4.3).
- Study dropouts decline further participation OR are **not** answering the daily headache question at least 3 days per week at least 4 weeks consecutively OR are non-adherent for at least 4 weeks consecutively, but are **not** answering the daily headache question at least 3 days per week during the weeks of non-adherence. For this group missing data will be imputed based on data from the control group using reference-based multiple imputations,[16,17] applying a copy increments in reference procedure. We will use the so-called copy increments in reference (CIR) procedure[18,19]. In the CIR procedure the intervention group's imputed outcomes after dropping out from the study mimic (parallel) the gradient from the control group.
- For sensitivity analyses we will use multiple imputations based on data of the entire data set, because we assume that patients dropping out will have already treatment benefits, because this has been observed in available data from M-sense Active. Therefore, this procedure also reflects a conservative approach. Multiple imputations based on the data of the intervention dropouts will not be considered as sensitivity analyses, because we expect this group to be too small for a robust multiple imputation procedure.

### 10.4.2 Dropouts in the control group

Dropouts in the control group (randomized to the control app) can only be study dropouts, because the control app does not include interventions.

Study dropouts are defined as patients who decline further participation OR are **not** answering the daily headache question at least 3 days per week at least 4 weeks consecutively. For this group missing data will be imputed based on data from the control group using reference-based multiple imputations,[16,17] assuming missing at random (MAR)[18,19].

### 10.4.3 Missing Data

Data completeness in the diary will be considered on a weekly basis and data are defined as missing if the daily headache question has not been answered at least 3 days per week during a respective week. Missing data for the diary will be imputed on the week-level. Missing data in questionnaires, will be imputed based on the measurement time point. For missing data imputation a multiple imputation procedure will be used (regression based multiple imputation method stratified by treatment group and type of disease (chronic or episodic migraine) with five imputed datasets.

Imputation model: imputations for continuous data will be generated with the predictive mean matching method (pmm). The following variables will be used as predictors in the imputation process for missing values at later time points: headache days, migraine days, age, gender, education level, duration of migraine disease, quality of life (HIT-6), self-efficacy (HMSE-G-SF), headache attributed burden (HALT-30), and migraine-specific health literacy.

In case the respective information for the classification of the headache days into migraine days, days with tension type headaches and other headaches has not been documented, we will use a conservative approach and assume a migraine day. Therefore, we will replace these missing values with migraine days in both groups, for the intervention phase as well as for the baseline phase.

## 10.5 ANALYSIS FOR THE EFFECTIVENESS OF THE APP

### 10.5.1 Primary analysis of the primary outcome

The analysis for the primary endpoint (migraine days week 9-12) will be conducted using an analysis of covariance (ANCOVA) with the treatment group (intervention/control) as fixed factor and baseline migraine days (during 28 days) and type of migraine disease (chronic vs. episodic migraine = stratification variable during randomization) as covariates.

The analyses will be based on the ITT population and handling drop out and missing data are defined in chapter 10.4.

From the ANCOVA model, estimated means and 95% confidence intervals, standard errors and the p-value for the group comparison will be obtained. The significance level is set at 0.05 (two-sided).

### 10.5.2 Sensitivity analyses of the primary outcome

The analysis of the primary outcome as described in 10.5.1 will be repeated:

- for the ITT as described above using an extended ANCOVA that includes also those variables as covariates, where clinically relevant baseline differences between the two groups (M-sense Migräne app, control app) were observed,
- for the ITT replacing missing data for study dropouts using multiple imputations,
- based on the FAS population.

Furthermore, a GLM (repeated-measure ANCOVA) will be fitted to the data based on the ITT to compare the two groups (M-sense Migräne app, control app) with respect to changes in the number of headache days per month over time (using data on a weekly bases from week 1-12). The model will include the treatment (M-sense Migräne app and control app) and time as fixed main effects, an interaction term for treatment by time, and the baseline headache days as well as type of migraine disease (chronic vs. episodic) as covariates.

All sensitivity analyses for the primary outcome will be considered explorative.

### 10.5.3 Moderators and Subgroup Analyses

Moderators on the primary outcome will be analyzed by using an analysis of variance (ANOVA) including an interaction term of the moderator and treatment condition. This will provide information on the importance of each moderator on the treatment effect (p-values and explained variance). We will use the following baseline variables as moderators:

- Gender (male/female/other)
- Baseline migraine days (low vs. high based on median split)
- Type of migraine disease (chronic/episodic migraine)
- Duration of migraine disease (short vs. long based on median split)

In addition to that, we will display for each subgroup means and confidence intervals. These analyses will be considered explorative.

### 10.5.4 Secondary outcomes

Secondary outcomes, that are considered continuous data, will be analyzed with an ANCOVA similar to the procedure for the primary outcome. A logistic regression analysis or Chi Square Test will be used for the comparison of the two treatment conditions for the dichotomous outcome responder. The analyses of secondary outcomes are summarized in Table 2 and will be considered explorative.

## 10.6 ANALYSIS OF ADHERENCE TO THE STUDY INTERVENTION

Adherence to the study intervention will be only analyzed in the group with M-sense Migräne because the control group has no app intervention. The adherence to the study intervention will be presented graphically over time as percentages of adherent patients per week. The analyses details are given in Table 3.

## 10.7 ANALYSIS OF SAFETY

Based on the safety population all safety variables will be tabulated (in total and per treatment group for adverse events). We will display the number of patients with serious adverse events, suspected adverse reactions, and serious suspected adverse reactions, and the number of patients who withdraw from the study due to safety concerns. Categorical variables are reported using frequency and percentage, continuous variables are reported by giving descriptive measures (mean, standard deviation/standard error, quartiles, and range). Groups will be compared by Chi-square or Wilcoxon rank test.

# 11 SOFTWARE

SAS for Windows, Version 9.4 (SAS Institute, Cary, NC, USA)

R Version 3.6.2

Stata

SPSS 26.0 (SPSS Inc., Chicago, USA)

**Table 2. Secondary** outcome analyses for comparisons between both groups

| Outcomes | Time of measurement | Refers to... | Scale | Possible values | Interpretation | Analysis method | Analysis population |
|---|---|---|---|---|---|---|---|
| Migraine days per month | daily | week 1-4, week 5-8 | continuous | 0-28 | higher values indicate higher disease burden | ANCOVA* | ITT |
| Headache days per month | daily | week 1-4, week 5-8, and week 9-12 | continuous | 0-28 | higher values indicate higher disease burden | ANCOVA* | ITT |
| Responder rate: at least 30% reduction of migraine days per month compared to baseline | daily | week 9-12 | dichotomous | Yes, no | higher values indicate more responders | Binary logistic regression* | ITT |
| Moderate/severe headache days per month | daily | week 1-4, week 5-8, and week 9-12 | continuous | 0-28 | higher values indicate higher disease burden | ANCOVA* | ITT |
| Days with acute headache medication intake | daily | week 1-4, week 5-8, and week 9-12 | continuous | 0-28 | higher values indicate higher medication use | ANCOVA* | ITT |
| Days with triptans intake | daily | week 1-4, week 5-8, and week 9-12 | continuous | 0-28 | higher values indicate higher medication use | ANCOVA* | ITT |
| Migraine specific quality of life (HIT-6) | week 12 | week 9-12 | continuous | 36-78 | higher values indicate lower quality of life | ANCOVA* | ITT |
| Self-efficacy (HMSE-G-SF) | week 12 | after 12 weeks | continuous | 6-42 | higher values indicate lower impairment | ANCOVA* | ITT |
| Headache attributed burden (HALT-30) | week 12 | 30 days | continuous | ≥0 | higher values indicate higher disease burden | ANCOVA* | ITT |
| Migraine-specific health literacy: item prophylaxis literacy | week 12 | after 12 weeks | continuous | 1-4 | higher values indicate lower health literacy | ANCOVA* | ITT |
| Migraine-specific health literacy: disease literacy, | week 12 | after 12 weeks | continuous | 1-4 | higher values indicate lower health literacy | ANCOVA* | ITT |
| Migraine-specific health literacy: trigger literacy | week 12 | after 12 weeks | continuous | 1-4 | higher values indicate lower health literacy | ANCOVA* | ITT |
| Number of patients with concomitant activity/activities against headache (all and specific) | daily | week 1-4, week 5-8, week 9-12 | dichotomous | Yes, no | | $Chi^2$ | ITT |
| Study dropouts | | week 1-4, week 5-8, and week 9-12, week 1-12 | dichotomous | Yes, no | | $Chi^2$ | ITT |
| **Safety measurements** | | | | | | | |
| Counts of serious adverse events | week 4, 8, and 12 | week 1-4, week 5-8, week 9-12 | continuous | ≥0 | higher values indicate higher number of serious adverse events | Wilcoxon rank test | ITT |
| Number of patients with at least one serious adverse event | week 4, 8, and 12 | week 1-4, week 5-8, week 9-12 | dichotomous | Yes, no | | $Chi^2$ | ITT |

* with treatment group (fixed factor) and respective baseline value (if applicable) as covariate

**Table 3.** Descriptive outcomes for the group with M-sense Migräne

| Outcomes | Time of measurement | Refers to… | Scale | Possible values | Interpretation | Analysis method | Analysis population |
|---|---|---|---|---|---|---|---|
| Percentage of patients adherend to app use | weekly | weeks 1-12 | dichotomous | Yes, no | higher percentages indicate better adherence | descriptively | ITT |
| **Safety measurements** | | | | | | | |
| Number of patients with at least one suspected adverse reaction | week 4, 8, and 12 | week 1-4, week 5-8, week 9-12 | dichotomous | Yes, no | | descriptively | Safety population |
| Duration of suspected adverse reaction | week 4, 8, and 12 | week 1-4, week 5-8, week 9-12 | continuous (Minutes, hours, days, weeks) | ≥0 | higher values indicate longer duration | descriptively | Safety population |
| Counts of suspected adverse reactions | week 4, 8, and 12 | week 1-4, week 5-8, week 9-12 | continuous | ≥0 | higher values indicate a higher number of suspected adverse reactions | descriptively | Safety population |

# 12 REFERENCES


1. Committee for Medicinal Products for Human Use. ICH E9 (R1) addendum on estimands and sensitivity analysis in clinical trials to the guideline on statistical principles for clinical trials. In: European Medicines Agency; 2020.
2. Headache Classification Committee of the International Headache Society (IHS) The International Classification of Headache Disorders, 3rd edition. *Cephalalgia.* 2018;38(1):1-211.
3. Roesch A, Dahlem MA, Neeb L, Kurth T. Validation of an algorithm for automated classification of migraine and tension-type headache attacks in an electronic headache diary. *J Headache Pain.* 2020;21(1):75.
4. Newsenselab GmbH. *Systematic Data Analysis of M-sense Active User Data.* Berlin2020.
5. Diener HC, Tassorelli C, Dodick DW, et al. Guidelines of the International Headache Society for controlled trials of preventive treatment of migraine attacks in episodic migraine in adults. *Cephalalgia.* 2020;40(10):1026-1044.
6. Tassorelli C, Diener HC, Dodick DW, et al. Guidelines of the International Headache Society for controlled trials of preventive treatment of chronic migraine in adults. *Cephalalgia.* 2018;38(5):815-832.
7. Kosinski M, Bayliss MS, Bjorner JB, et al. A six-item short-form survey for measuring headache impact: the HIT-6. *Qual Life Res.* 2003;12(8):963-974.
8. Ware JE, Jr., Bjorner JB, Kosinski M. Practical implications of item response theory and computerized adaptive testing: a brief summary of ongoing studies of widely used headache impact scales. *Med Care.* 2000;38(9 Suppl):II73-82.
9. Graef JE, Rief W, French DJ, Nilges P, Nestoriuc Y. German Language Adaptation of the Headache Management Self-Efficacy Scale (HMSE-G) and Development of a New Short Form (HMSE-G-SF). *Headache.* 2015;55(7):958-972.
10. Steiner TJ, Lipton RB, Al Jumah M, et al. The Headache-Attributed Lost Time (HALT) Indices: measures of burden for clinical management and population-based research. *The Journal of Headache and Pain.* 2018;19(1):12.
11. Springer Healthcare IME. Migraine management. Springer Healthcare IME. https://migraine-management.ime.springerhealthcare.com/pro-tool/. Published 2020. Accessed 19-11-2020, 2020.
12. Stewart WF, Lipton RB, Kolodner KB, Sawyer J, Lee C, Liberman JN. Validity of the Migraine Disability Assessment (MIDAS) score in comparison to a diary-based measure in a population sample of migraine sufferers. *Pain.* 2000;88(1):41-52.
13. Diener HC, Schorn CF, Bingel U, Dodick DW. The importance of placebo in headache research. *Cephalalgia.* 2008;28(10):1003-1011.
14. CONSORT. http://www.consort-statement.org. Published 2019. Accessed 20-11-2020, 2020.
15. Moher D, Schulz KF, Altman DG. The CONSORT statement: revised recommendations for improving the quality of reports of parallel-group randomized trials. *AnnIntern Med.* 2001;134(8):657-662.
16. Cro S, Morris TP, Kenward MG, Carpenter JR. Reference-based sensitivity analysis via multiple imputation for longitudinal trials with protocol deviation. *Stata J.* 2016;16(2):443-463.
17. Carpenter JR, Kenward MG. *Multiple imputation and its application.* 1st ed. Chichester, West Sussex: John Wiley & Sons; 2013.
18. Carpenter JR, Roger JH, Kenward MG. Analysis of longitudinal trials with protocol deviation: a framework for relevant, accessible assumptions, and inference via multiple imputation. *J Biopharm Stat.* 2013;23(6):1352-1371.
19. Leurent B, Gomes M, Cro S, Wiles N, Carpenter JR. Reference-based multiple imputation for missing data sensitivity analyses in trial-based cost-effectiveness analysis. *Health Econ.* 2020;29(2):171-184.
