# Supplementary material for: A Prescribed Digital Health App and Number of Migraine Days: A Randomized Clinical Trial
Source: JAMA Netw Open. 2025 Jul 1;8(7):e2517708. doi: 10.1001/jamanetworkopen.2025.17708 (PMC12215570; doi:10.1001/jamanetworkopen.2025.17708)
Supplement: Supplement 2. — eTable 1. Results from sensitivity analyses with multiple imputation eTable 2. Results from sensitivity analyses with complete case analysis (FAS analysis) eTable 3. Results of sensitivity analysis with ANCOVA eTable 4. Dropout analysis eFigure. Screenshots of the “M-sense Migräne” app eMethods 1. Theoretical basis of applied behavior change theories and techniques in the app eMethods 2. Handling of dropouts and missing data imputation [file jamanetwopen-e2517708-s002.pdf]

Supplementary Online Content

Pach D, Lysk S, Heinz P, et al. A prescribed digital health app and number of migraine days: a randomized clinical trial. *JAMA Netw Open*. 2025;8(7):e2517708.
doi:10.1001/jamanetworkopen.2025.17708

eTable 1. Results from sensitivity analyses with multiple imputation

eTable 2. Results from sensitivity analyses with complete case analysis (FAS analysis)

eTable 3. Results of sensitivity analysis with ANCOVA

eTable 4. Dropout analysis

eFigure. Screenshots of the "M-sense Migräne" app

eMethods 1. Theoretical basis of applied behavior change theories and techniques in the app

eMethods 2. Handling of dropouts and missing data imputation

eReferences.

This supplementary material has been provided by the authors to give readers additional information about their work.

© 2025 Pach D et al. *JAMA Network Open*.

eTable 1. Results from sensitivity analyses with multiple imputation

	**Coefficient**	**Lower bound of 95% CI**	**Upper bound of 95% CI**	**p-value**	**Adj. mean inter-vention**	**Lower bound of 95% CI**	**Upper bound of 95% CI**	**Adj. mean control**	**Lower bound of 95% CI**	**Upper bound of 95% CI**
Intercept	4.025	2.144	5.906	< 0.0001	··	··	··	··	··	··
Intervention group	0.415	-1.342	2.171	0.58	6.447	5.402	7.492	6.032	5.029	7.036
Baseline migraine days	0.329	0.074	0.585	0.02	··	··	··	··	··	··
Stratification group Episodic migraine	-1.094	-2.523	0.336	0.13	··	··	··	··	··	··

CI: confidence interval

eTable 2. Results from sensitivity analyses with complete case analysis (FAS analysis)

	**Coefficient**	**Lower bound of 95% CI**	**Upper bound of 95% CI**	**p-value**	**Adj. mean inter-vention**	**Lower bound of 95% CI**	**Upper bound of 95% CI**	**Adj. mean control**	**Lower bound of 95% CI**	**Upper bound of 95% CI**
Intercept	4.808	2.713	6.904	< 0.0001	··	··	··	··	··	··
Intervention group	0.195	-0.575	0.965	0.62	6.472	5.545	7.400	6.277	5.366	7.189
Baseline migraine days	0.274	0.164	0.385]	< 0.0001	··	··	··	··	··	··
Stratification group episodic migraine	-1.365	-3.056	0.326	0.11	··	··	··	··	··	··

CI: confidence interval

eTable 3. Results of sensitivity analysis with ANCOVA

Repeated-measures ANCOVA (Sensitivity analyses, according to SAP section 10.5.2) based on data imputed by reference-based imputation was performed.In this analysis, migraine days per month (month 1 to 3), adjusted for baseline migraine days and type of migraine disease were addressed. Month was entered in a linear way.

	**Coefficient**	**Lower bound of 95% CI**	**Upper bound of 95% CI**	**p-value**
Intervention group	0.838	0.293	1.384	0.003
Month	-0.439	-0.660	-0.218	0.0001
Intervention group:month	-0.475	-0.803	-0.147	0.005

CI: confidence interval

© 2025 Pach D et al. *JAMA Network Open*.

eTable 4. Dropout analysis

In this analysis, the numbers of study dropouts were compared between treatment groups across week 1-4, 5-8, 9-12 separately, and overall 1-12. The study dropouts (dichotomous) were addressed with Fisher's exact tests.

week	Number of dropouts intervention group	Number of dropouts Control group	p-value (Fisher test)
1-4	18	23	0.51
5-8	42	39	0.81
9-12	42	44	0.91
1-12	44	44	1.00

© 2025 Pach D et al. *JAMA Network Open*.

eFigure. Screenshots of the “M-sense Migräne” app

App diary

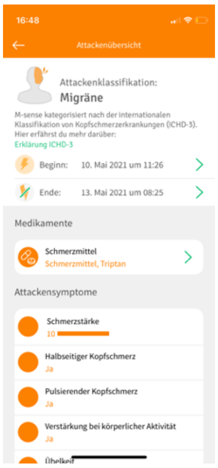

Symptom entry & attack classification

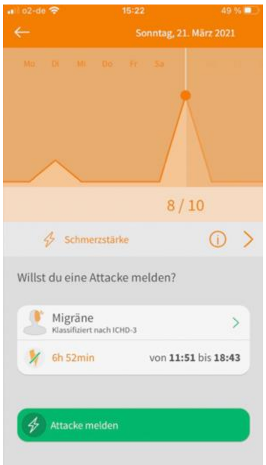

Weekly attack timeline with pain intensity

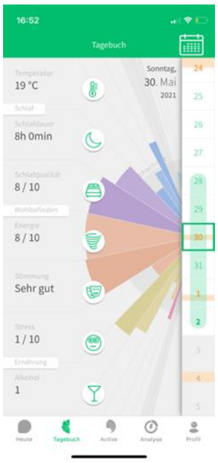

Trigger diary

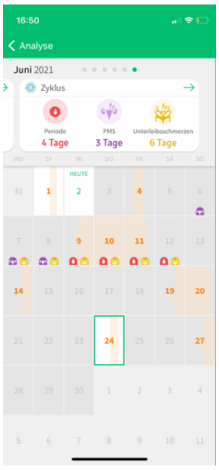

Calendar overview (menstrual cycle)

Report & data analysis

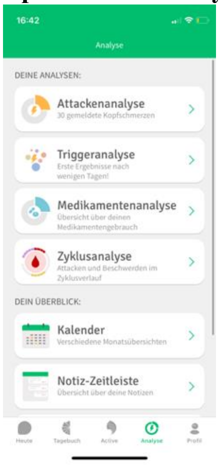

Overview screen of the analysis function

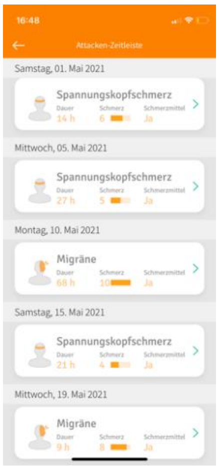

Attack timeline (with attack classification)

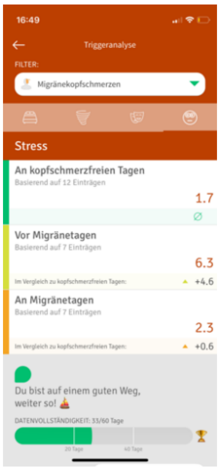

Trigger analysis (example: analysis of stress)

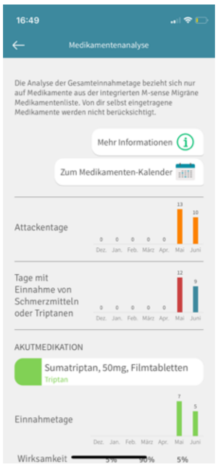

Analysis of medication intake

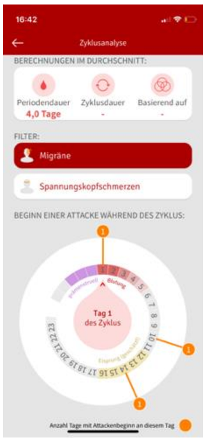

Cycle analysis for menstrual migraine

© 2025 Pach D et al. *JAMA Network Open*.

“Active” self-management

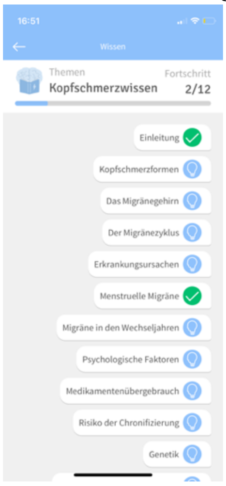

Overview screen of knowledge lessons

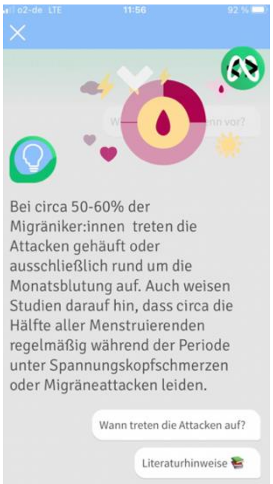

Chatbot integrated into the knowledge lessons

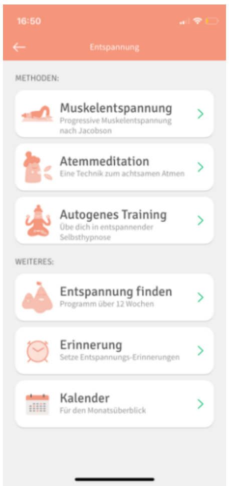

Overview screen relaxation exercises

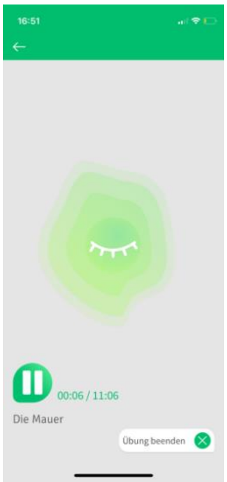

Relaxation exercise (audio file)

Behaviour Change Techniques

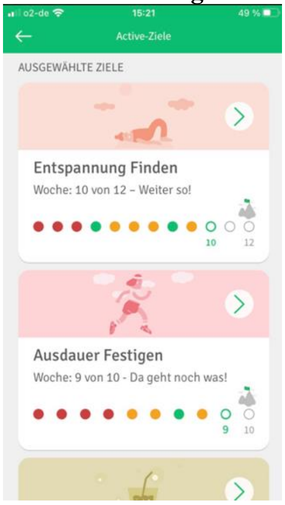

Goal setting with weekly feedback (here relaxation and endurance sports)

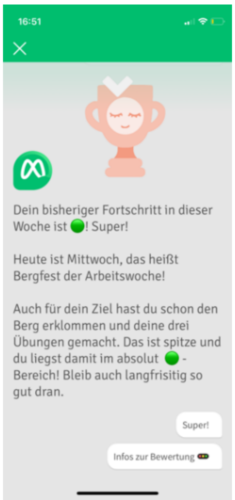

Rewards and integrated chatbot to give personalized feedback and boost self-efficacy

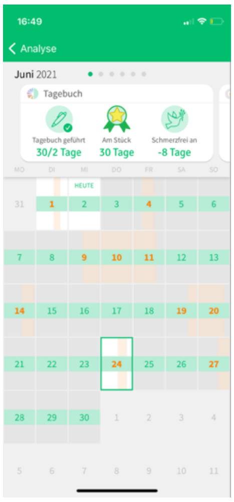

Self-monitoring of diary usage (with a reward for continuous tracking)

© 2025 Pach D et al. *JAMA Network Open*.

eMethods 1. Theoretical basis of applied behavior change theories and techniques in the app

The app placed strong emphasis on fostering intention building by enhancing self-efficacy (via patient stories and positive feedback) and developing positive outcome expectancies (e.g. by personalized knowledge topics) as described in the Health Action Process Approach by Schwarzer et al.[1] For the goal-pursuit phase, the app incorporated action planning (e.g. training plans with reminders) and feedback.

To enhance app-engagement and promote self-management, several behavior change techniques[2] have been implemented, including: "goal-setting (behavior)" (e.g. maintaining a regular sleep schedule or engaging in regular exercises), "action planning", "self-monitoring of behavior" (e.g. calendar); "feedback on discrepancies between current behavior and goal setting", "prompts/cues", "instruction on how to perform a behavior", "rewards" and "verbal persuasion to boost self-efficacy".

eMethods 2. Handling of dropouts and missing data imputation

In the case of study dropouts, missing data was imputed based on the distribution of observations in the control group using reference-based multiple imputation employing a copy increments in reference (CIR) procedure[3]. The CIR approach within the reference-based multiple imputation was used in the main analysis of the primary outcome, as well as in the analysis of migraine days from week 1 to 8 and the responder rate. Reference-based multiple imputation was based on the baseline value of the outcome and the stratification variable (type of migraine) in an ANCOVA model. The uncertainty measures were computed by using jackknife samples. As conditional mean imputation needs normal distribution of the outcome variable, a square root transformation of the weekly numbers was used to stabilize the variance. Afterwards, back-transformation was done and the numbers over 4 weeks were summed up. Sensitivity analyses of this analysis were three-fold: first, standard 5-fold multiple imputation with chained equations (MICE)[4] was used to account for missing values in both treatment groups, second, a larger set of pre-specified variables were included into the reference-based multiple imputation, and third, a complete-case analysis based on the full analysis set was performed.

The imputation model for the MICE procedure included the following variables: type of disease, age, gender, education, years since migraine onset (demographic and baseline values), migraine days, headache days, moderate/severe headache days, days with acute headache medication use (including triptans), days with triptan use, days with acute headache medication use (without triptans) (baseline value and weekly value in week 1 to 12), quality of life (HIT-6), self-efficacy (HMSE-G-SF), headache attributed burden (HALT-30), and the three migraine-specific health literacy scores (at baseline and follow-up measurement in week 12).[4]

Secondary analyses included all longitudinal repeated observations in migraine days over time. Linear mixed-effects models with random intercepts were used for these analyses. In these models, time was

© 2025 Pach D et al. *JAMA Network Open*.

considered as a continuous variable, and potential interaction effects with the treatment group were evaluated.

The analysis of the secondary outcomes, number of headache days, number of moderate/severe headache days, number of days with acute headache medication use, was based on standard 5-fold multiple imputation on a weekly level.

eReferences

1. Schwarzer R, Lippke S, Luszczynska A. Mechanisms of health behavior change in persons with chronic illness or disability: the Health Action Process Approach (HAPA). *Rehabil Psychol* 2011;56(3):161-70. doi: 10.1037/a0024509

2. Michie S, Richardson M, Johnston M, et al. The behavior change technique taxonomy (v1) of 93 hierarchically clustered techniques: building an international consensus for the reporting of behavior change interventions. *Ann Behav Med* 2013;46(1):81-95. doi: 10.1007/s12160-013-9486-6 [published Online First: 2013/03/21]

3. Wolbers M, Noci A, Delmar P, et al. Standard and reference-based conditional mean imputation. *Pharm Stat* 2022;21(6):1246-57. doi: 10.1002/pst.2234 [published Online First: 20220519]

4. van Buuren S, Groothuis-Oudshoorn K. mice: Multivariate Imputation by Chained Equations in R. *Journal of Statistical Software* 2011;45(3):1 - 67. doi: 10.18637/jss.v045.i03

© 2025 Pach D et al. *JAMA Network Open*.
