# Supplementary material for: A Prescribed Digital Health App and Number of Migraine Days: A Randomized Clinical Trial
Source: JAMA Netw Open. 2025 Jul 1;8(7):e2517708. doi: 10.1001/jamanetworkopen.2025.17708 (PMC12215570; doi:10.1001/jamanetworkopen.2025.17708)
Supplement: Supplement 3. — Data Sharing Statement [file jamanetwopen-e2517708-s003.pdf]

Data Sharing Statement

Pach. A Prescribed Digital Health App and Number of Migraine Days. *JAMA Netw Open*.
Published July 01, 2025. doi:10.1001/jamanetworkopen.2025.17708

Data
Additional Information: DRKS00024174
Data available: Yes
Data types: Deidentified participant data
How to access data: daniel.pach@charite.de
When available: With publication

Supporting Documents
Document types: Informed consent form, Other (please specify)
Additional Information: statistical analysis plan
How to access documents: daniel.pach@charite.de
When available: With publication

Additional Information
Who can access the data: Anonymized participants data will be available to researchers upon reasonable request to the corresponding author.
Types of analyses: any purpose
Mechanisms of data availability: after approval of a proposal
